# The burden of typhoid fever in low- and middle-income countries: A meta-regression approach

**DOI:** 10.1371/journal.pntd.0005376

**Published:** 2017-02-27

**Authors:** Marina Antillón, Joshua L. Warren, Forrest W. Crawford, Daniel M. Weinberger, Esra Kürüm, Gi Deok Pak, Florian Marks, Virginia E. Pitzer

**Affiliations:** 1 Department of Epidemiology of Microbial Diseases, Yale School of Public Health, New Haven, Connecticut, United States of America; 2 Department of Biostatistics, Yale School of Public Health, New Haven, Connecticut, United States of America; 3 Department of Statistics, University of California Riverside, Riverside, California, United States of America; 4 International Vaccine Institute, Seoul, Republic of Korea; Oswaldo Cruz Foundation, BRAZIL

## Abstract

**Background:**

Upcoming vaccination efforts against typhoid fever require an assessment of the baseline burden of disease in countries at risk. There are no typhoid incidence data from most low- and middle-income countries (LMICs), so model-based estimates offer insights for decision-makers in the absence of readily available data.

**Methods:**

We developed a mixed-effects model fit to data from 32 population-based studies of typhoid incidence in 22 locations in 14 countries. We tested the contribution of economic and environmental indices for predicting typhoid incidence using a stochastic search variable selection algorithm. We performed out-of-sample validation to assess the predictive performance of the model.

**Results:**

We estimated that 17.8 million cases of typhoid fever occur each year in LMICs (95% credible interval: 6.9–48.4 million). Central Africa was predicted to experience the highest incidence of typhoid, followed by select countries in Central, South, and Southeast Asia. Incidence typically peaked in the 2–4 year old age group. Models incorporating widely available economic and environmental indicators were found to describe incidence better than null models.

**Conclusions:**

Recent estimates of typhoid burden may under-estimate the number of cases and magnitude of uncertainty in typhoid incidence. Our analysis permits prediction of overall as well as age-specific incidence of typhoid fever in LMICs, and incorporates uncertainty around the model structure and estimates of the predictors. Future studies are needed to further validate and refine model predictions and better understand year-to-year variation in cases.

## Author summary

Typhoid fever is a bacterial enteric infection that continues to pose a considerable burden to the 5.5 billion people living in low- and middle-income countries (LMICs). We developed and validated a model incorporating widely available indicators of economic and social development and the environment to estimate the burden of typhoid fever across LMICs. Our analysis uses all available data to estimate the incidence of typhoid in key age groups, which is important for the design and implementation of optimal vaccination strategies, and it identifies regions of the world that have the most uncertainty in typhoid incidence. Across all LMICs, we estimated that the expected number of typhoid fever cases per year is 17.8 million (95% CI: 6.9–48.4 million). We also present the probability that incidence surpasses the criteria for low, medium, high, and very high incidence in each country, which could help guide policy in the face of uncertainty.

## Introduction

Typhoid fever has been estimated to cause between 9.9 and 24.2 million cases and 75,000–208,000 deaths per year [1–3]. Typhoid fever is caused by infection with *Salmonella enterica* serovar Typhi, a gram-negative bacterium that invades the body via the small intestines and colonizes macrophages in the reticuloendothelial system, from where it is shed into the bloodstream [4,5]. Symptoms of the resulting disease typically include prolonged fever, frontal headache, malaise and marked loss of appetite, sometimes accompanied by abdominal pains, nausea, and (in severe cases) intestinal perforation and neurological complications [6]. Symptoms typically subside in 7–21 days, but mortality is estimated to occur in 1–5% of hospitalized patients [7–9]. In a small percentage of cases, the bacteria may also colonize the gall bladder, leading to a chronic carrier state [6].

Data on the incidence of typhoid fever are scarce in low- and middle-income countries (LMICs). The symptoms of typhoid fever resemble those of many other significant febrile diseases, precluding straightforward estimates of typhoid incidence [10,11]. Recent estimates have relied on key expert assumptions, primarily geographical groupings that coincide with UN development regions or pre-determined epidemiological regions [1,2,12]. The degree to which incidence may be attributable to geography as well as to indicators of poverty and socioeconomic circumstances remains mostly unexamined [3]. Considering that typhoid incidence may vary both between and within countries, it is necessary to identify potential predictors of incidence that facilitate interpolation across LMICs, where the disease is suspected to remain endemic.

Furthermore, variation in the age distribution of typhoid fever across settings is not well understood. Cases tend to be concentrated in younger age groups in settings with higher transmission and distributed more equally among different ages in low-transmission settings [1,2,12]. However, recent studies have cast doubt on the generality of these age patterns in relation to overall incidence [10,13–15]. Identifying predictors of the age distribution of typhoid fever is of particular salience to the design and implementation of optimal vaccination strategies.

We explored the potential contribution of demographic, environmental, and socioeconomic indicators that serve as candidate predictors of the age-specific incidence of typhoid fever. We used a data-driven approach to predict the mean and variance in age-specific incidence while accounting for uncertainty in the underlying model structure, and validated our predictions against out-of-sample data.

## Methods

### Data sources

We carried out a literature search to identify population-based studies that reported incidence of culture-confirmed typhoid fever for the period of 1980–2014. We excluded all hospital-based or clinic-based studies that did not constitute exhaustive surveillance of typhoid in a well-defined population. Further details of the literature search are presented in the S1 Text.

We gathered data on possible predictors of typhoid fever incidence from publicly available databases, aiming to identify indicators of environmental characteristics and socioeconomic development for all LMICs. Predictors were chosen for their ubiquity and relevance to water-borne disease transmission in consultation with typhoid experts. Table 1 lists the predictors included in this analysis and the source of data; S1 Text provides more details. The predictors’ values were matched as closely as possible to the time and location of each incidence study.

**Table 1:** Summary statistics for the candidate predictors included in the predictive model.

Covariate	Resolution	Mean and range in estimation sample	Mean and range in TSAP* sample	Mean and range in prediction sample	Source
Population density: pop/km^2^ †	1/24 x 1/24 degree	2,091 (17–47,024)	1,205 (46–11,592)	7.06 (0–98,928)	[16]
GDP per capita, 2005 international dollars †	1x1 degree	$1,780 ($500-$5,509)	1,130 (841–1,608)	$6,909 (694–53,836)	[17]
Gini coefficient	National	41 (31–59)	42 (37–50)	39 (29–57)	[18]
Access to improved source of water ‡	Subnational, national	35 (2–90)	25 (3–66)	61 (1–100)	[19,20]
Access to improved source of sanitation ‡	Subnational	38 (2–96)	17 (3–63)	57 (0.36–98)	[19,20]
Years of education for women over age 20 †	Subnational	4.7 (1.9–10.3)	3.8 (0.5–9.8)	1.7 (0.6–10.7)	[18,19]
% roads paved ‡	National	40 (10–87)	4 (4–28)	47 (1.8–91)	[18]
% population living on <$2/day ‡	National	59 (18–93)	78 (52–93)	25 (0.05–95)	[18]
Prevalence of stunting ‡	Subnational, national	42 (6–60)	34 (18–51)	17 (0.02–82.7)	[18,19]
Number of major floods 1985–2011 ^2^	50m^2^	21 (4–99)	11 (1–25)	3.7 (0–32)	[21]
HIV prevalence ‡	National	0.958 (0.013–6.2)	2.46 (0.3–6)	1.42 (0.1–28.8)	[22]
Water stress †	½ x ½ degree	1.7 (0.0021–6.8)	1.17 (0.07–3.46)	1.37 (0.10–10)	[23]

* TSAP: Typhoid Fever Surveillance in Africa Program

^†^ Log transformed values were used. Geometric means were reported in this case.

^‡^ Logistic transformed values were used.

We validated our model predictions against previously unpublished data from the Typhoid Fever Surveillance in Africa Program (TSAP, see S2 Table), which consisted of passive, population-based surveillance in 9 of 13 sites in 10 countries across sub-Saharan Africa [24,25]. We extracted predictor data for the TSAP studies from the same databases used for the estimation sample (Table 1).

### Model framework

**Fig 1:**
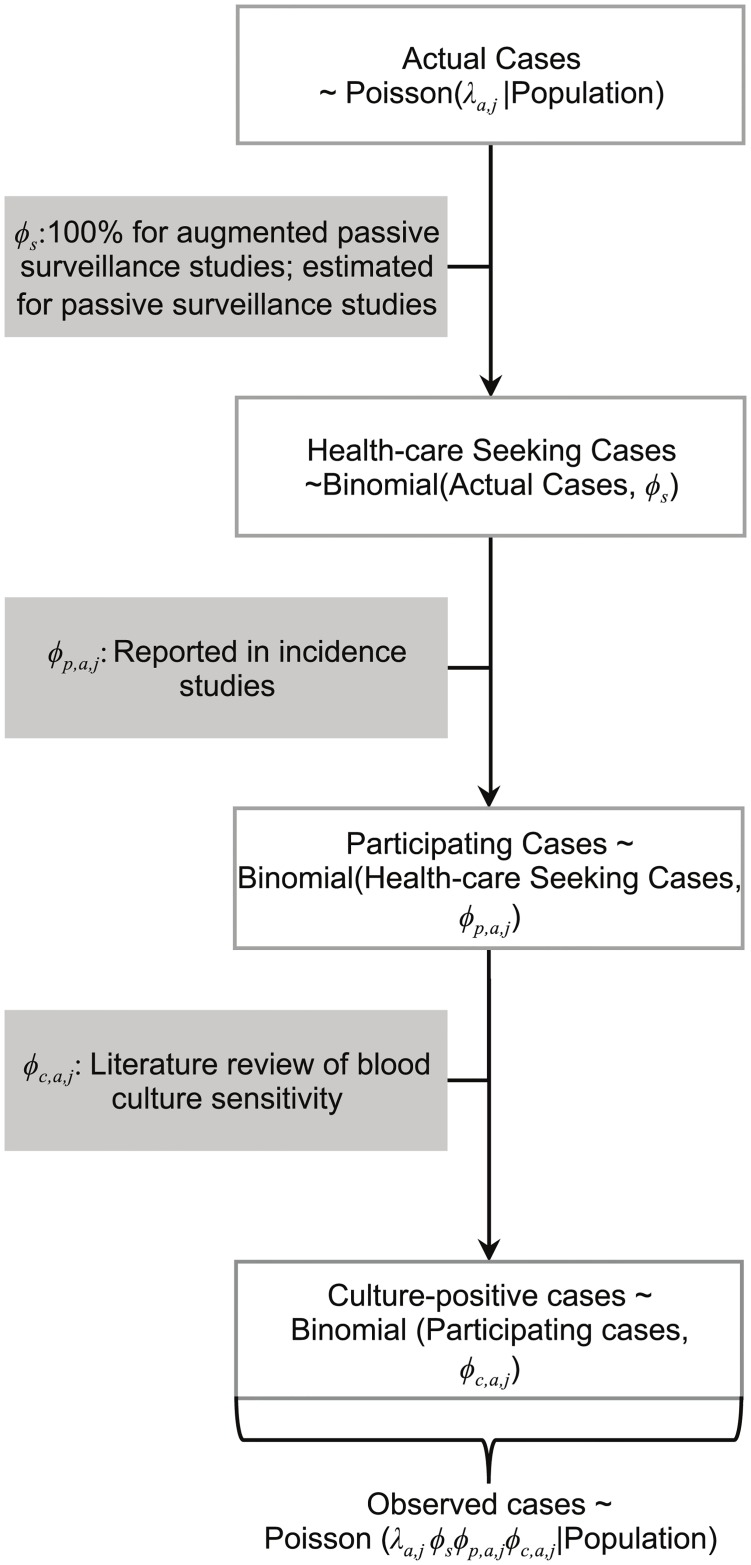
Flowchart of the disease and observation process reflected in our data. The top of the flowchart consists of all cases of typhoid fever in the population, which is what we are ultimately interested in estimating. However, three intervening (observation) processes result in a considerable difference between the “actual” cases of typhoid fever and the observed cases of typhoid fever. In all, the observation process is made up of the type of surveillance employed in each study (*ϕ*_*s*_), the participation rate of patients seen at each of the study clinics (*ϕ*_*p*,*a*,*j*_, adjusted for age *a* and study site *j*), and the sensitivity of blood culture used to confirm typhoid infection (*ϕ*_*c*,*a*,*j*_, adjusted for age *a* and study *j*). We modeled the observed cases as representing the successful trials of a binomial process (the detection process), where the number of trials is Poisson distributed (with a rate parameter equal to the “true” incidence of typhoid cases, *λ*_*a*,*j*_, at age *a* and study site *j*); thus, the number of observed cases is a thinned Poisson distribution with rate parameter equal to the product of the disease rate and the probability of a case being detected at each step of the observation process. We used Bayesian priors to account for the observation process and adequately estimate the underlying disease process.

The observed data on typhoid fever incidence result from both a disease process and an observation process. We aimed to take into account both of these processes in our modeling strategy, as illustrated in Fig 1.

#### Disease process

We employed a generalized linear mixed-effects model with a log link function to characterize the true underlying incidence of typhoid (*λ*_*a*,*j*_) in setting *j* and age group *a*: <2 years, 2–4 years, 5–14 years, ≥15 years and older. We estimated the age-specific incidence as a function of fixed effects (modeled by the inclusion of predictors for slope coefficients of the age variables) and location-specific random effects. The age groups were chosen based on the resolution of the available data and their salience in typhoid vaccine policymaking: current vaccines are licensed for children over 2 years of age, and some vaccine programs have employed school-based campaigns targeting children 5–14 years of age [26,27]. Observations from some sites were only available for a combination of age groups, so we adjusted our analysis where necessary to accommodate the lessened granularity of the observed age patterns (see S1 Text).

First, we considered a null model in which annual incidence was based on age-group fixed effects and random effects for each age group in each location. The intercept, *B*_*0*_, represents the incidence in the referent age group (designated as 5–14 years olds) in each setting, and the slopes *B*_*a*_ represent the incidence rate ratio between the three other age groups and the referent group. An offset equal to the log of person-time was included to adjust for the size of the population under observation:

$${\text{Actual Cases}}_{a,j}∼\text{Poisson}\left(\lambda_{a,j}{|\text{person}-\text{time}}_{a,j}\right)$$

$$\text{log}(\lambda_{a,j})=B_{0,j}+B_{a,j}+\text{log}\left({\text{person}-\text{time}}_{a,j}\right)$$

$$B_{0,j}=\beta_{0}+\alpha_{0,j}$$

$$B_{a,j}=\beta_{a}+\alpha_{a,j}$$

where *α*_0,*j*_ ~ MVN(0, *Σ*) with *Σ* as the covariance structure of the random effect terms. *B*_*a*,*j*_ = 0 for 5–14 year olds.

To test the assumption that typhoid fever incidence correlates with indicators of environmental characteristics and socioeconomic development, we evaluated whether including additional covariates (*X*_*j*_) to model both the intercept and the slope improved our ability to predict the incidence of typhoid fever:

$${\text{Actual Cases}}_{a,j}∼\text{Poisson}\left(\lambda_{a,j}{|\text{person}-\text{time}}_{a,j}\right)$$

$$\text{log}(\lambda_{a,j})=B_{0,j}+B_{a,j}+\text{log}\left({\text{person}-\text{time}}_{a,j}\right)$$

$$B_{0,j}=\beta_{0}+\gamma X_{j}+\alpha_{0,j}$$

$$B_{a,j}=\beta_{a}+\eta_{a}X_{j}+\alpha_{a,j}$$

where *γ* and *η*_*a*_ are the effect sizes corresponding to each predictor for the intercept and slope, respectively; again, *α*_0,*j*_ ~ MVN(0, *Σ*) and *B*_*a*,*j*_ = 0 for 5–14 year olds. Variable selection for the predictors was based on the spike-and-slab method described below.

#### Observation process

**Fig 2:**
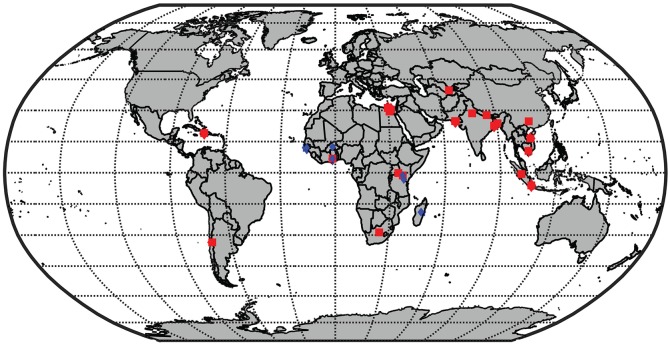
Map of the location of studies in our dataset. Studies used in the estimation sample are depicted in red and the studies used in the validation sample are depicted in blue. The studies in the validation sample come from the Typhoid Fever Surveillance in Africa Program (TSAP).

Three additional sub-processes that impact our observed data were taken into account (Fig 2, details in S1 Text). First, we adjusted for the difference in case ascertainment between active and passive surveillance. We assumed active surveillance would be capable of identifying all cases and estimated a fixed effect for the relative ascertainment under passive surveillance (see S1 Text). Second, we included the participation rate of patients at each location (by age, if possible) as a fixed parameter based on the reported proportion of patients meeting the case definition who had blood drawn for diagnosis. Third, we adjusted for the sensitivity of blood culture to detect typhoid infection. We estimated the test sensitivity as a function of age group for each location using strong prior distributions informed by a meta-analysis of the relationship between sample volume and blood culture sensitivity (See S1 Text).

#### Model selection and parameter estimation

We used a Bayesian framework for model estimation, which allowed us to incorporate prior information on diagnostic test sensitivity, as described above. We used a stochastic search variable selection algorithm employing spike-and-slab priors to estimate the fixed effects of the predictors while incorporating uncertainty in model structure (see S1 Text) [28,29]. To assess convergence on the space of predictor combinations, we ran two chains, one initiated with a null model and one initiated with a saturated model; if each covariate was selected for inclusion with approximately equal chance in each chain, we concluded that the algorithm had converged. The model was fit using the JAGS (Just Another Gibbs Sampler) software, version 3.4.0, in conjunction with MATLAB 2014b via the MATJAGS interface [30,31].

### Model validation

We sought to validate the predictive ability of our model in two ways: using a leave-k-out validation method and by comparing model predictions to out-of-sample data from the recent TSAP studies [24,32]. For the leave-k-out validation, we randomly partitioned the dataset into seven sets of three locations each. We fit the model to data from six of the seven sets of locations, and used the fitted model to predict the incidence in the seventh set. We re-sampled the model seven times, excluding one of the groups each time. To assess the improvement in model fit, we compared the covariance in the predictions produced by the null model and the model with predictors, as well as predictions from the leave-k-out validation.

### Posterior model predictions

We drew 1,000 samples from the estimated model in order to obtain posterior predictions of the incidence rate across all countries classified as lower income, lower-middle income, or upper-middle income at least once in the period of 2011–2015 by the World Bank. We mapped the median estimates of the predicted incidence by age using a map resolution of 0.1 degrees. We capped the predicted incidence at 10,000 per 100,000 person-years. To obtain estimates of the incidence for each region, we took the population-weighted sum of the estimated incidence over the raster surface. To characterize the uncertainty in the incidence, we calculated the proportion of the posterior predictive sample in each of four incidence categories: <10, 10-<100, 100-<500, and ≥500 cases per 100,000 person-years, designated as low, medium, high, and very high incidence.

## Results

We identified 32 studies from 22 sites located in 14 countries (Fig 2); these studies are detailed in S1 Table. We extracted data on case counts, person-time of observation, and details of study design. In total, the studies observed 2,668 cases of typhoid in 3,329,183 person-years of observation. The validation dataset observed 140 cases of typhoid fever during 212,312 person-years of observation.

### Predictor selection and age-specific incidence estimates

**Fig 3:**
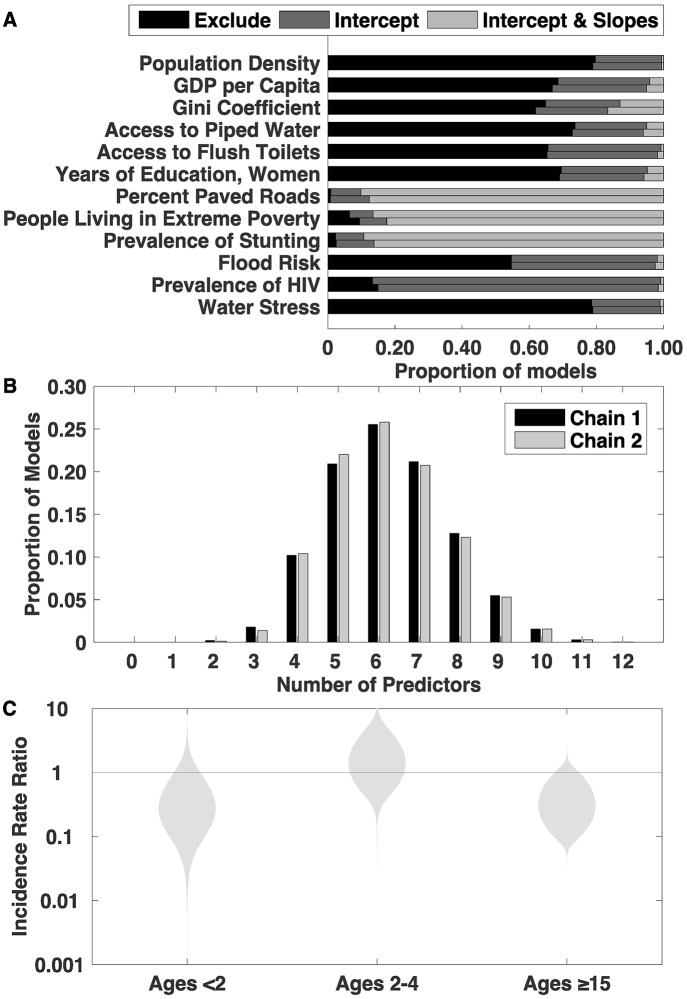
Model summary. A) The posterior marginal probability that each variable was excluded from the model (black) or included as a predictor of the intercept (dark grey) or intercept and slope (light grey) is shown for two chains. Our stochastic search variable selection algorithm could include variables either as a predictor of the intercept (the incidence in 5–14 year olds) or as a predictor of the intercept as well as the slopes (the incidence rate ratios between the other age groups and the referent age group of 5–14 year olds). B) Distribution of the average number of covariates in the model. Chain 1 was initiated using a model that included all the covariates as predictors of the main effect, while chain 2 was initiated as the null model. The null model was never sampled, implying that the models including at least one predictor better described the data than the null model. C) Posterior distributions of age-specific incidence rate ratios between the referent age group (5–14 years of age) and other age groups: <2 years, 2–4 years, ≥15 years old.

We evaluated twelve potential predictors of typhoid fever incidence (Table 1). Fig 3A shows the posterior distribution of the probability of inclusion for each predictor. Both chains provided equivalent probabilities of variable inclusion as well as equivalent distributions for the size of the underlying model (Fig 3B), indicating that our algorithm converged.

No single covariate was included in all models. The percent of roads paved, prevalence of stunting, and percent of the population living in extreme poverty were the most sampled covariates (present in 99%, 97%, and 91% of all models in both chains); in almost all models in which they were present, the covariates were useful to predict both the overall incidence as well as the age-specific incidence rate ratios in each setting. HIV prevalence and flood risk were the next most sampled predictors (present in 85% and 45% of all models in both chains), but these indices were only useful to predict the overall incidence. Indices for income inequality (Gini coefficient), access to flush toilets, and GDP per capita were sampled in just over a third of all models, while the rest of the predictors were each included in 21–30% of all models. In total, the models had a median of six predictors, and 95% of models had between three and nine predictors; the null model was never sampled (Fig 3B).

**Fig 4:**
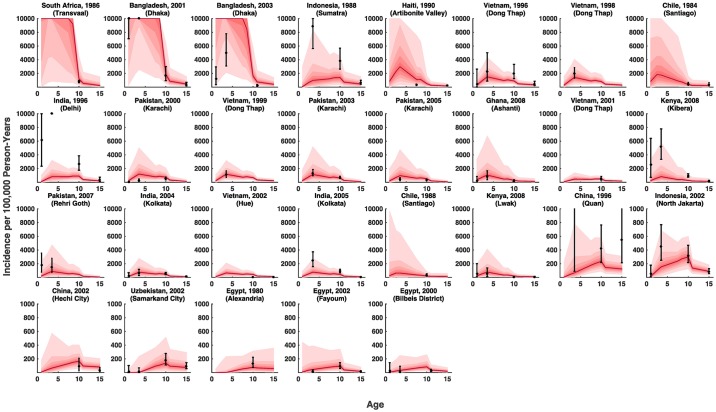
Observed versus predicted age-specific incidence rates. Sites are labeled by location and year, and plots are ordered by decreasing overall model-predicted incidence. The red line and regions represent the model fits—median and 95% credible interval of the expected incidence estimated by the joint posterior distribution of model parameter (excluding study specific random effects and the impact of the observation process). The black symbols are the observed incidence with the 95% credible intervals after adjusting for the observation process: surveillance type (active/augmented passive versus passive surveillance), the participation rate, and blood culture sensitivity. Only studies that reported age-specific incidence are featured here.

The model estimated a lower incidence rate (on average) among <2 year olds and ≥15 year olds, and a slightly higher incidence rate among 2–4 year olds compared to 5–14 year olds (Fig 3C). Furthermore, the model reproduced the heterogeneity in age-specific incidence (Fig 4). A weak relationship between overall incidence and the shape of the age distribution is evident: a higher overall incidence is associated with a peak in incidence among children 2–4 years of age instead of a more uniform burden across different ages, with a slight peak among 5–14 year olds in lower incidence settings.

### Model validation and predictions

**Fig 5:**
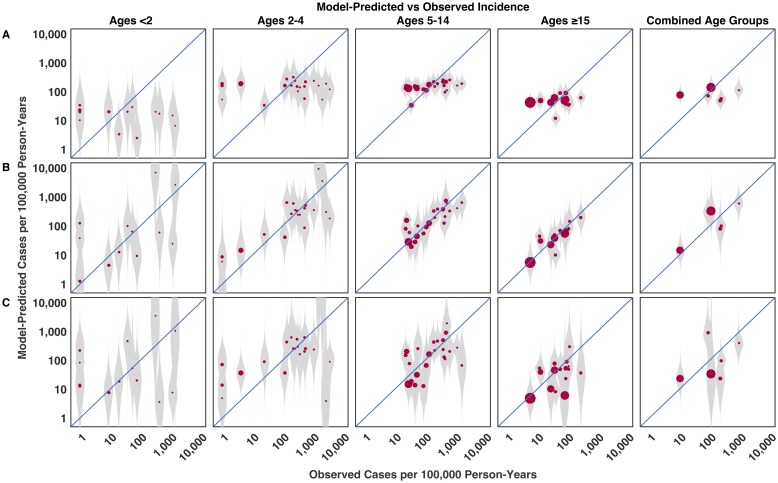
Observed versus model-predicted incidence. (A) Posterior predictions from the null model, which only adjusts for age and the observation process. (B) Posterior predictions from the model using fixed effects for the predictors. (C) Leave-3-out validation results. The gray markers represent the density of model-predicted posterior distributions of incidence, while the red dots represent the median posterior predicted incidence. The size of the red circular markers is proportional to the number of person-years of observation in each study. All predictions are of the mean incidence and were generated using only the fixed-effect terms of the model, and hence do not account for unmeasured location-specific differences, e.g. in healthcare-seeking behavior.

The posterior incidence predictions are shown in Fig 5 for the null model, the model with predictors, and the leave-k-out validation. Including the predictors improved the model fit, although the models tended to underestimate the incidence in <5 year olds in high incidence settings and overestimate it in low incidence settings. The model including random effects explained the residual variance and provided a good fit to the data (S3 Fig). Importantly, leave-k-out validation shows that most out-of-sample credible intervals contained the observed incidence (Fig 5C). Moreover, estimating the models using subsets of the data (all but 3 observations) would yield similar profiles in terms of the variables selected for inclusion and the size of the model (S4 and S5 Figs).

**Fig 6:**
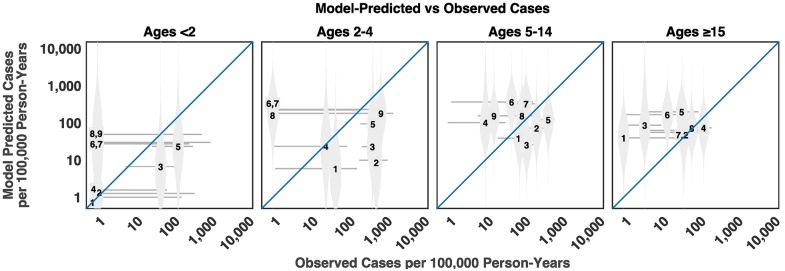
Out-of-sample validation. The observed versus predicted incidence of typhoid fever is plotted for studies in the Typhoid Fever Surveillance in Africa Program (TSAP) using a model estimated from previously published data identified in our literature review. The numbers represent the median posterior predicted incidence for each TSAP site: 1- Nioko II, Burkina Faso. 2 –Polesgo, Burkina Faso. 3 –Ashanti Akim North, Ghana. 4 –Bandim, Guinea Bissau. 5 –Kibera, Kenya. 6 –Antananarivo, Madagascar. 7 –Imerintsiatosika, Madagascar. 8 –Moshi rural, Tanzania. 9 –Moshi urban, Tanzania. The gray markers represent the density of model-predicted posterior distributions of incidence. The gray horizontal lines represent 95% confidence intervals for the observed incidence.

Model validation against the TSAP data performed well for some sites, but showed large variance in posterior estimates of incidence, as well as notable overestimates of incidence in several locations (Fig 6). The 95% credible intervals (CIs) overlapped with the observed incidence in some locations, but not others. However, the 95% CI of the observed incidence also showed a large amount of uncertainty in directly measured incidence estimates (sometimes spanning three orders of magnitude). No clear geographic pattern emerged to distinguish between observations that did and did not match model predictions. The model successfully predicted the incidence in Kibera, Kenya (the site of a previous study) for <15 year olds, as well as locations for which there was no previous data, e.g. Bandim (Guinea Bissau), rural Moshi (Tanzania) for ≥2 year olds, Polesgo (Burkina Faso) for all ages except 2–4 year olds, and Nioko II in Burkina Faso for <15 year olds. Successful prediction in more than one setting, as well as more than one age group, indicates that our models were able to capture some of the within-country heterogeneity in incidence.

**Fig 7:**
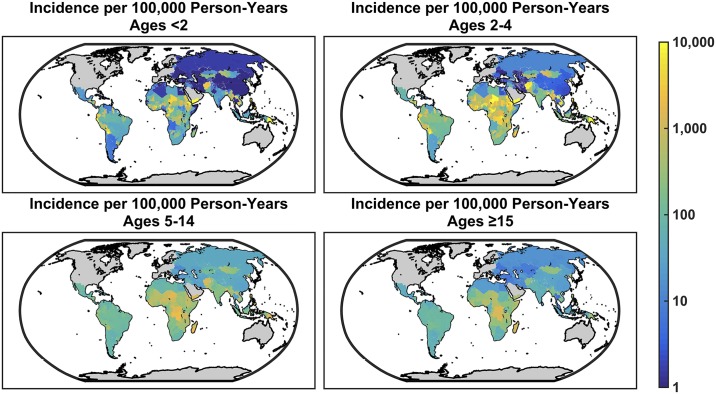
Model-predicted age-specific incidence per 100,000 person-years. The median posterior predicted incidence per 100,000 person-years in each of the age groups (<2 years, 2–4 years, 5–14 years, and ≥15 years) is mapped for all low- and middle-income countries (LMICs) with a resolution of 0.1 degrees.

Given the environmental and socioeconomic composition of LMICs, our model predicts that typhoid fever incidence should be highest in parts of Central Africa, Turkmenistan and Uzbekistan in Central Asia, as well as Mongolia and western China (Fig 7). Incidence is predicted to be highest in 2–4 year olds or 5–14 year olds, but the age of peak incidence varies from place to place (Fig 7). The incidence is lower in children <2 years of age and adults in most settings.

Approximately 6 billion people lived in all LMICs in 2015. We estimated that the expected number of typhoid fever cases per year is 17.8 million across all LMICs (95% CI: 6.9–48.4 million) (Table 2). According to our analysis, almost 40% of all cases occur in sub-Saharan Africa (7.2 million, 95% CI: 2.2–30.2 million), although the uncertainty around our estimates is considerable.

**Table 2:** Total cases and incidence for the Global Burden of Disease regions and subregions made up of low- and middle-income countries. Total cases are shown in millions and incidence is per 100,000 person-years.

	Cases	Incidence
**All LMICs**	**17.8 (6.9, 48.4)**	**293 (111, 794)**
		
**Central Europe, Eastern Europe, and Central Asia**	**0.1 (0.02, 0.6)**	**28 (7, 166)**
Central Asia	0.05 (0.01, 0.5)	55 (12, 541)
Central Europe	0.01 (0.003, 0.06)	21 (4, 100)
Eastern Europe	0.03 (0.01, 0.13)	16 (4, 65)
**Latin America and Caribbean**	**1.0 (0.2, 3.9)**	**169 (32, 642)**
Andean Latin America	0.4 (0.04, 2.1)	704(80, 3751)
Caribbean	0.02 (0.004, 0.05)	47(12, 166)
Central Latin America	0.3 (0.07, 1.3)	120 (30, 512)
Southern Latin America	0.04 (0.01, 0.2)	61 (15, 276)
Tropical Latin America	0.2 (0.04, 1.1)	89 (18, 517)
**North Africa and Middle East**	**2.6 (0.5, 5.7)**	**557 (100, 1208)**
		
**Sub-Saharan Africa**	**7.2 (2.2, 30.2)**	**762 (230, 3208)**
Central Sub-Saharan Africa	1.7 (0.4, 8.4)	1459 (371, 6984)
Eastern Sub-Saharan Africa	2.4 (0.8, 11.3)	620(213, 2921)
Southern Sub-Saharan Africa	0.1 (0.04, 0.4)	149 (57, 571)
Western Sub-Saharan Africa	2.8 (0.7, 11.2)	753 (198, 3075)
**Southeast Asia, East Asia, and Oceania**	**2.21 (0.7, 6.8)**	**108 (36, 334)**
Southeast Asia	1.3 (0.4, 5.3)	217 (88, 571)
East Asia	0.5 (0.1, 1.7)	33 (9, 122)
Oceania	0.4 (0.03, 0.5)	5454 (397, 6576)
**South Asia**	**3.6 (1.5, 9.4)**	**204 (64, 851)**

### Geographic heterogeneity

Although South Asia has the second largest case count among the regions, the region has the third highest expected incidence rate after sub-Saharan Africa and North Africa/Middle East (Table 2). Considerable heterogeneity in the expected incidence exists within each region, as well. Within sub-Saharan Africa, Central Africa has the highest expected incidence and Southern Africa has the lowest expected incidence. The incidence in Andean Latin America outpaces the incidence in other parts of Latin America by a factor of six or more. Oceania has the highest incidence of any sub-region in the world (Table 2).

### Quantifying uncertainty

**Fig 8:**
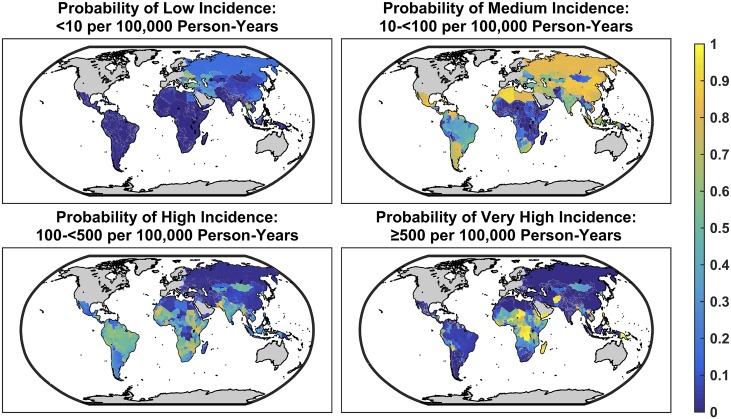
Probability that each location falls into one of four incidence categories: <10, 10-<100, 100-<500, and ≥500 cases per 100,000 person-years, designated as low, medium, high, and very high incidence, respectively.

Our model allowed us to quantify the probability that each continent and its sub-regions had a total incidence that fell into one of four incidence categories: <10 cases per 100,000 person-years (“low incidence”), 10-<100 cases per 100,000 person-years (“medium incidence”), 100-<500 cases per 100,000 person-years (“high incidence”) and ≥500 cases per 100,000 person-years (“very high incidence”) (Table 3). All sub-regions of sub-Saharan Africa except Southern Sub-Saharan Africa have a high probability of being in the highest incidence category. North Africa and the Middle East have the second highest probability of being in the very high incidence category, while South Asia falls into the high incidence category. Latin America and the region of Southeast Asia, East Asia and Oceania have a 67% and 56% probability, respectively, of belonging in the high-incidence category, but geographic heterogeneity within both regions is considerable. While most parts of Latin America are likely to experience medium incidence, Central Latin America is most likely to experience high incidence, and Andean Latin America is most likely to experience very high incidence. Similarly, while East Asia is most likely to experience medium incidence, Southeast Asia is most likely to experience high incidence, and Oceania is most likely to experience very high incidence (Table 3 and Fig 8).

**Table 3:** Uncertainty in incidence estimates for the Global Burden of Disease regions and subregions made up of low- and middle-income countries.

	Percent of posterior sample in each incidence category			
	Low (<10 per 100,000 person-years)	Medium (10-<100 per 100,000 person-years)	High (100-<500 per 100,000 person-years)	Very high (≥500 per 100,000 person-years)
**Central Europe, Eastern Europe, and Central Asia**	7	87	6	<1
Central Asia	<1	77	20	3
Central Europe	14	83	2	<1
Eastern Europe	23	76	1	<1
**Latin America and Caribbean**	<1	27	67	7
Andean Latin America	<1	5	37	59
Caribbean	1	90	9	<1
Central Latin America	<1	40	57	3
Southern Latin America	<1	75	25	<1
Tropical Latin America	<1	57	40	3
**North Africa and Middle East**	<1	2	41	57
**Sub-Saharan Africa**	<1	<1	26	74
Central Sub-Saharan Africa	<1	<1	7	93
Eastern Sub-Saharan Africa	<1	<1	38	62
Southern Sub-Saharan Africa	<1	24	72	4
Western Sub-Saharan Africa	<1	<1	26	74
**Southeast Asia, East Asia, and Oceania**	<1	44	56	<1
Southeast Asia	<1	12	79	10
East Asia	3	92	4	<1
Oceania	<1	<1	4	96
**South Asia**	<1	5	91	4

## Discussion

We developed a meta-regression framework incorporating widely available indicators of economic and social development and the environment to estimate the incidence of typhoid fever across LMICs, as well as the concomitant uncertainty. We identified predictors that explain a substantial amount of heterogeneity in the incidence of typhoid fever, which significantly improved predictions of incidence across all age groups and for school-aged children in particular. This analysis represents substantial progress over existing models for the incidence of typhoid fever by allowing for estimation of variation in typhoid incidence both within and between countries. Given the limited data available, the credible intervals around the model predictions are appropriately large in parts of the world where typhoid surveillance is weak or non-existent. Although considerable uncertainty remains, an additional strength of our analysis is that we calculate the probability that incidence surpasses the criteria for low, medium, high, and very high incidence in each country, which could help guide policy in the face of uncertainty.

Our results provide insights into the likely predictive power of widely available risk factors for typhoid incidence; however, these should not be interpreted as providing inference on the causes of typhoid transmission. First, most of the covariate data is available at the national or sub-national administrative unit, potentially failing to capture the epidemiological dynamics that operate at smaller scales. Second, these indices might be adequate proxies for the causal factors that drive disease incidence in some locations, but not across all LMICs. For instance, although contaminated drinking water has been established as the vehicle of transmission in numerous outbreak investigations, the proportion of the population using piped water is not a particularly helpful metric for predicting typhoid incidence in LMICs compared to other indicators of development and health, e.g. percent of roads paved, the population living in extreme poverty, or the prevalence of stunting and HIV [33–36]. The relatively weak association between typhoid incidence and the percent of the population with access to flush toilets or piped water may reflect that these indicators do not capture the microbial quality of water in the home [37]. Other estimates of typhoid incidence have relied on indicators of improved sources of water and sanitation in order to make assumptions about country-to-country and sub-national variation in typhoid fever incidence, but these studies performed limited or no assessment of the validity of these predictors for typhoid incidence [2,3]. Notably, no other study has examined the comparative utility of such a wide variety of indicators for predicting typhoid fever incidence.

Our estimate of the overall incidence of typhoid fever is similar to those published previously, but it reflects considerably greater uncertainty [1,2,12]. Previous studies assumed that each country experienced the mean incidence in its UN region [2,12] or median incidence in its GBD super region [1]. When we ran the model assuming regional hyperpriors for the location-specific random effects of the intercept and the slopes (rather than a single global hyperprior for the random effects), we found that regional hyperpriors did not differ significantly from each other or from the global hyperprior for the regional-level effects (S4 Table). Furthermore, none of the previous studies attempted to perform within-sample or out-of-sample validation [1,3,12,38]. Finally, it should also be noted that previous studies did not adjust for the apparent difference in incidence reported in passive versus “augmented passive” or active surveillance studies.

Past estimates of the incidence of typhoid fever in different age groups did not account for differential diagnostic procedures amongst age groups (in particular blood volume used for culture confirmation). It is important to consider the degree to which the observed age distribution of disease, particularly the lower incidence often observed in <5 year olds, could be attributed to the relationship between test sensitivity, the amount of blood drawn for diagnosis, and age. By simultaneously estimating the overall incidence, the incidence rate ratios between different age groups, and the observation process, we have allowed the data to drive our estimates of age-specific incidence, rather than relying on assumptions derived from a subset of studies.

However, we did not adjust for all differences in diagnostic procedures across studies. For example, while most studies limited eligible participants to those with a fever of three days or more, at least two of the studies enrolled all febrile children <5 years old regardless of the duration of fever [14,39]. Furthermore, blood cultures were carried out manually in older studies, whereas most recent studies have used an automated blood culture apparatus such as BacTec or BacT/ALERT that enhances the sensitivity of the culture to detect *S*. Typhi. The random-effect terms should account for at least some of these differences, as well as unmeasured differences in healthcare-seeking behavior, but were not factored into our model predictions, since such information is not available for all LMIC settings.

After carrying out a rigorous, statistically sound analysis of the available data on typhoid incidence, sizable uncertainty around our incidence estimates remains. The uncertainty in the incidence rate ratio (IRR) among <5 year olds and the covariance in the IRR between the <2 and 2–4 year olds outpaces the between and within-group variance in the other groups (S3 Table). Although numerous studies did not sample adults, the variance around the IRR for adults was the smallest of any age group. This indicates that future studies should focus on the incidence in children <5 years of age in order to refine the uncertainty around typhoid incidence estimates.

We strongly emphasize the need to consider the uncertainty in estimated incidence, in addition to the point estimates. Whereas past estimates designated countries into low, medium, and high incidence categories without any discussion on the potential for misclassification, our Bayesian framework allows us to present estimates of the probability that the incidence falls into each of four categories of incidence (Table 3, Fig 8). In addition, our stochastic search variable selection approach allowed us to incorporate both parameter and structural uncertainty in our estimates, which reflects our uncertainty not just in model coefficients but also in the combination of covariates that best explains incidence. We believe that this is a more honest appraisal of the extant data on typhoid incidence and its predictors, and that it could serve as a useful guide for policymakers on two matters: 1) what is the potential value of bolstering surveillance in order to refine our understanding of typhoid incidence, and 2) under the current level of uncertainty, what are the possible outcomes of different interventions?

Past estimates indicated that a large number of settings are predicted to fall into the high typhoid incidence category, previously defined as ≥100 cases per 100,000 person-years [2,40]. Our analysis suggests that this masks a formidable amount of the heterogeneity present in higher-incidence contexts; for instance, within Southeast Asia, East Asia, and Oceania, Southeast Asia is most likely to belong to the 100-<500 cases per 100,000 person-years incidence category, but Oceania is likely to have a higher incidence. We recommend further delineation of a “very high typhoid incidence” category to describe settings with an incidence of ≥500 per 100,000 person-years, which may motivate different or additional strategies for the control of typhoid fever in these settings. Our predictions also highlight potential within-country variation in the incidence of typhoid, which can be useful to policymakers in developing control strategies targeted at particular regions of a country.

It is clear that the incidence of typhoid fever in Africa is not yet well understood. Out-of-sample validation of the model against data from nine TSAP sites showed that the model has mixed success in predicting incidence for locations outside the estimation sample. The incidence in the <5 year age group rural site in Moshi, Tanzania, and in both sites in Madagascar were all over-estimated. Model predictions for Kibera, Kenya and Ashanti Akim North, Ghana were more consistent with the observed data; however, there were previous studies from these two sites that were used to estimate the model. This suggests that there was some consistency in the incidence of typhoid over time, which our model was able to capture. Moreover, we draw attention to the potential levels of typhoid fever in regions where typhoid has received limited or no attention in the literature, such as Central Africa, Central Asia, and Oceania, and Latin America.

The current study modeled the long-term incidence (technically speaking, the mean annual incidence) of typhoid fever instead of predicting the number of cases in any given year. As we lack high-quality data in almost all LMIC settings, predicting year-to-year variation in the incidence of typhoid fever would be considerably more difficult, but would likely lead to even greater uncertainty in the incidence of typhoid in any given year. Due to limited surveillance capacity for typhoid fever over prolonged periods of time, our estimates of the spatial distribution of typhoid incidence reflect only the spatial distribution of risk factors, rather than temporal processes of spatial contagion suggested by phylogeographic studies [35,41].

Furthermore, we do not account for temporal variations in incidence associated to emergent properties of the pathogen or its transmission dynamics, such as the recent outbreaks of typhoid reported across eastern and southern Africa [42–44], possibly related to the emergence of the H58 haplotype [45]. A few studies in our sample were carried out in the same place in different years using similar surveillance protocols, and in these places, observed incidence was rarely significantly different from one year to another, with the exception of Dong Thap, Vietnam, where there was a small downward trend in incidence (Fig 4). While some hospital-based time series attest to the stability of typhoid incidence over periods >10 years [46–48], others highlight the potential for abrupt changes in incidence [48]. Our model assumes typhoid is endemic, and therefore may not perform as well in Africa, which appears to be more prone to epidemics of typhoid (as well as cholera) [49]. Rather, these incidence estimates should be interpreted as the potential endemic burden of typhoid under current conditions. Nevertheless, it is important to consider how variation in typhoid incidence over time and space may impact the design and implementation of optimal control strategies for typhoid fever.

Our analysis achieved two main objectives: 1) to identify widely available predictors of typhoid fever incidence; 2) to point out places in the world that have the most uncertainty in typhoid incidence, thereby motivating future studies into the scale and spatiotemporal distribution of typhoid fever in these areas. However, many LMICs have limited capacity for typhoid surveillance. The model we present provides a validated means of predicting typhoid incidence in countries with limited or no typhoid surveillance data based on widely available indicators. Understanding and predicting the burden of typhoid fever is an essential first step in motivating the need for better control measures, including typhoid conjugate vaccines.

## Supporting information

S1 Text Detailed methods. (DOCX)

S1 Table Incidence studies used to estimate the parameters of the prediction model. Incidence rates (and 95% confidence intervals) per 100,000 person-years. Incidence rates shown are not adjusted for participation rate, surveillance type, or blood culture sensitivity.
(DOCX)

S2 Table Incidence studies used to validate our model. Incidence rates (and 95% confidence intervals) per 100,000 person-years. Incidence rates shown are not adjusted for participation rate, surveillance type, or blood culture sensitivity.
(DOCX)

S3 Table Random effect variance-covariance matrix. (DOCX)

S4 Table Posterior distributions for a model with 2-level hyperpriors to estimate random effects. We ran a null model assuming regional hyperpriors for the location-specific random effects (in addition to a global hyperprior) using two schemes to group countries: continents and the Global Burden of Disease (GBD) Regions for 2015. We discovered that regional hyperpriors would not differ significantly from global hyperpriors. Further, we note that there are only data for two locations in the Americas, making a separate hyperprior for this region unnecessary.
(DOCX)

S1 Fig Blood culture sensitivity. A) Studies that estimate blood culture sensitivity in bone-marrow culture-confirmed patients for typhoid fever [50–55]. B) Our predictions of sensitivity of blood cultures performed with a range of sample volume 2-15mL.
(EPS)

S2 Fig Posterior distributions for intercept and slope coefficients. Distribution of the covariate coefficients for the intercept and each of the slopes are shown. The symbol size corresponds to the proportion of the models that included that covariate. The intercept describes the incidence rate for children 5–14 years of age, slope 1 describes the incidence rate ratio (IRR) between children <2 and 5–14, slope 2 describes IRR between children 2–4 and 5–14, and slope 3 describes the IRR between adults (≥15) and children 5–14 years of age.
(EPS)

S3 Fig Observed versus model-predicted incidence using location-specific random effects. Posterior predictions from the model using fixed effects for the predictors and location- and age-specific random effects. The gray markers represent the density of model-predicted posterior distributions of incidence, while the red dots represent the median posterior predicted incidence. The size of the circular markers represents the number of person-years of observation in each study.
(EPS)

S4 Fig Predictor selection under leave-k-out validation. To validate the model, we re-estimated the model seven times, each time leaving out the data from three locations to assess how well the model would estimate the incidence in those locations. Leave-3-out validation allowed us to evaluate the consistency with which spike-and-slab priors (our stochastic search variable selection algorithm) would include each variables as predictors of the intercept or of the intercept and the slopes.
(EPS)

S5 Fig Model size under leave-k-out validation. Validation also allowed us to evaluate the posterior number of predictors selected for the model. Chain 1 was initiated using a model that included all the covariates as predictors of the main effect, while chain 2 was initiated as the null model. The null model was never sampled, implying that the models including at least one predictor better described the data than the null model.
(EPS)
