# Supplementary material for: The burden of typhoid fever in low- and middle-income countries: A meta-regression approach
Source: PLoS Negl Trop Dis. 2017 Feb 27;11(2):e0005376. doi: 10.1371/journal.pntd.0005376 (PMC5344533; doi:10.1371/journal.pntd.0005376)
Supplement: S1 Text — (DOCX) [file pntd.0005376.s001.docx]

**Supplementary Text**

**A meta-regression approach to predicting the incidence of typhoid fever in low- and middle-income countries**

Marina Antillón^1*^, Joshua L. Warren^2^, Forrest W. Crawford^2^, Daniel M. Weinberger^1^, Esra Kürüm^3^, Gi Deok Pak^4^, Florian Marks^4^, Virginia E. Pitzer^1^

*^1^Department of Epidemiology of Microbial Diseases, Yale School of Public Health, New Haven, CT 06520-8034 USA*

*^2^Department of Biostatistics, Yale School of Public Health, New Haven, CT 06520-8034 USA*

*^3^Department of Statistics, University of California Riverside, Riverside, CA 92521 USA*

*^4^International Vaccine Institute, Seoul 151-818, Republic of Korea*

# **Literature search and data extraction**

A literature review was undertaken to identify population-based studies that reported incidence of culture-confirmed typhoid fever for the period of 1980-2014. PubMed and Web of Knowledge were searched in English, Spanish, and French. Our search keywords included "typhoid fever", "Salmonella Typhi", and "Salmonella" linked to studies relating to "surveillance", "burden", "incidence", or "vaccine trials." From vaccine trials, we took into account only the incidence reported for the control arm of the study. In addition, we searched the bibliographies of all previous global burden of typhoid fever studies [1–4].

Data were extracted from population-based studies that reported age-specific incidence as well as studies that only reported overall incidence. In addition to case counts and person-time of observation, we extracted information on whether surveillance was passive or active, the volume of blood collected for culture, and the participation rate for each age group (if available). Hospital-based studies were included if both outpatient and inpatient cases were reported and we could identify the catchment population size. While we believed that the ascertainment of cases in hospital-based studies could be low, these studies provided a lower bound on typhoid incidence in regions of the world for which we had little or no information and also helped inform estimates of the age distribution of typhoid in different places.

We found that although some Ministries of Health reported typhoid incidence each year (e.g. Chile, Lebanon, Brazil), these agencies did not detail the circumstances under which the cases were reported well enough to understand how the data were comparable to population-based studies in the scientific literature; therefore, we did not include these data.

In total, we identified 32 studies in 22 sites located in 14 countries, carried out in the period between 1980-2010 (Table S1). Fourteen of these studies employed active surveillance or “augmented passive” population-based surveillance (in which study team members visited households to ask about the occurrence of fever and encourage febrile participants to attend study clinics), four studies were passive hospital-based studies, and the other fourteen studies were passive population-based surveillance studies. All but three studies reported incidence for separate age categories or for some subset of the age groups in our analysis: <2 years, 2-4 years, 5-15 years, ≥15 years old. We extracted data to conform to these age categories as closely as possible, but when incidence could not be broken down into these categories, we included the observation as a combination of the age categories, as described in section 3 of this supplement.

# **Predictor data**

## ***Country-level data***

For each of the surveillance studies described above, country-level data was extracted from the World Bank's World Development Index to match the geographic location of the study, whether it took place in an urban or rural context, and the period of the study as closely as possible [5]. Specifically, we extracted indices of inequality (Gini coefficient), extreme poverty (headcount ratio: % households living with <$2/person per day), and infrastructure (% roads paved). When there was data for multiple years of the study, the predictor data was matched to the midpoint year of the study. Data was available for most countries from 1960-present, but many years were missing data. In these instances, values for the predictors associated with a study were estimated by linear interpolation. If only one value was available for any given predictor in the period 1980-present, that value was extracted for the study. In the event that any data were still missing, we took the average value of the given predictor in the corresponding World Bank income group in the year that the data was missing.

## ***Subnational data***

Three of the predictors in our model were based on subnational data: population density (persons per kilometer), gross domestic product (GDP) per capita (adjusted for Purchasing Power Parity), and flood risk (measured as the number of floods between 1985 and 2011).

***Population Density.*** We used population density maps with a resolution of 2.5 arc-minutes from the National Aeronautics and Space Administration's Socioeconomic Data and Applications Center (SEDAC)[6].

***Health and Women’s Education Data***. We used data compiled and harmonized from a variety of sources by Radboud University Nijmegen’s Global Data Lab. Using this database, we extracted the data aggregated by subnational region (usually the province level) for access to piped water, access to flush toilets, percent of children that are stunted, and the average number of years of education for women over the age of 20. Although these data were rich in terms of geographical detail, there was limited resolution in terms of temporal trends. For the indices of women’s education and the prevalence of stunting, we were able to use linear interpolation to estimate the values of the index in years for which there was little data. For sanitation and flush toilets, we used data from the WHO’s Joint Monitoring Programme (JMP) and the World Bank’s WDI database to impute the magnitude of change in these indices at the national level in order to extrapolate the values of these indices across time at the subnational level. For countries for which there was no subnational data, we used the national data from the WHO and the World Bank. If there was no national-level data for a country, we took the average value of the given predictor in the corresponding World Bank income group in the year that the data was missing.

***Economic Data.*** In order to capture subnational economic data, we used Geographically-based Economic data (G-Econ 4.0) available from Yale University (gecon.yale.edu) [7,8]. The dataset contains point data at 1-degree resolution for GDP (adjusted for Purchasing Power Parity) at four time points: 1990, 1995, 2000, 2005, as well as approximate population counts for each location.

We used these data to compute GDP per capita, and we used a linear interpolation of these data to create a raster file of GDP per capita. Geographic data were missing from some countries in some years; for these countries we assigned values equal to the GDP per capita reported to the World Bank's World Development Index (WDI) for the whole country for that year.

For the years 2005-2014, we used the national GDP per capita growth figures from the World Bank to project the subnational GDP estimates from 2005 to 2015. The drawback of this approach is that it assumes that the economy in the country grows homogenously; this may be inaccurate in countries with increasing economic disparities among different regions.

***Flood Risk and Water Stress.*** We used the flood risk maps at 0.5-degree resolution from the World Resource Institute[9]. We used the water stress maps at 0.5-degree resolution from the Water Footprint Network in collaboration with the University of Twente (Netherlands), which estimated the map of water scarcity for the period of 1996-2005[10].

## ***Data Processing***

***Rasterizing the country-level and subnational unit data.*** We used a shapefile in the public domain published by Natural Earth (www.naturalearthdata.com) of the political boundaries for each of 247 countries in the world compiled using data from the United Nations as well as the U.S. Central Intelligence Agency. We merged country-level data with this map and converted these data into a raster dataset of 0.1-degree resolution in order to combine it with the subnational-level data that we acquired in raster form. We also rasterized the maps representing flood risk (which were available as shapefiles of flood plains).

***Imputing spatial data.*** We used nearest-neighbor interpolation to impute values for these indicators for each of the typhoid surveillance study sites. Time-variation was not taken into account when imputing flood risk in each of the study sites, but we imputed the population density and GDP per capita according to the 5-year period closest to the year when the study took place.

***Prediction maps.*** Since our population density maps were of much higher resolution than the maps for GDP and flood risk, and population density and population counts in each cell could have a high impact on our final case counts, we downscaled our GDP and flood risk maps to a resolution of 0.1-degree via linear interpolation, and we up-scaled our population density maps to a resolution of 0.1 degree. The drawback of up-scaling the population density map was a slight loss of information, but we circumvented computational memory issues.

## ***Predictors in the training dataset vs. the prediction dataset***

We checked the range of the training set predictors in order to make sure that their distributions approximated the distributions of the covariates of the countries for which we wanted to provide predictions of typhoid incidence. A comparison of the predictor values used to fit the model and those used to make posterior predictions is found in Table 1 of the main text.

# **Model**

Our estimation model consists of two major components: one component models the latent disease process, and a second component models the observation process. We were therefore able to study and estimate the degree of variance between different contexts attributable to the underlying disease incidence model as opposed to the ascertainment process of the data.

## ***Model of disease process***

We employed a mixed effects model, where we tested predictors for both the intercept (***B_0,j_***, which estimates incidence for our referent age group, school-aged children 5-14 years old in location ***j***) and for the slope (***B_a_*_,_*_j_*** which estimates the incidence rate ratio between the referent age group and the other age groups of interest *a*, including children <5 years of age and adults in location *j*). In the null model, we have one fixed effect ($\boldsymbol{\alpha}_{\boldsymbol{0}}$and $\boldsymbol{\beta}_{\boldsymbol{a}}$) for each of the age groups, and a location-specific random effect for the intercept, $\boldsymbol{\alpha}_{\boldsymbol{j}}$, which allows us to adjust for the correlation between repeat studies within the same country. Alternative models were tested as well to assess whether region-level random effects could explain the variation.

We performed a stochastic search variable selection method in order to test whether additional fixed effects corresponding to our set of possible predictors, ***X_j_***, multiplied by coefficients for the effect sizes corresponding to each predictor (***γ*** and ***η_a_*** for the intercept and slope models, respectively) could help explain trends in the typhoid burden data.

$$\text{Actual Cases}_{\boldsymbol{a,j}}\boldsymbol{\sim}\text{Poisson}\left( \boldsymbol{\lambda}_{\boldsymbol{a,j}} | \text{person-years}_{\boldsymbol{a,j}} \right)$$

$$\log\left( \boldsymbol{\lambda}_{\boldsymbol{a,j}} \right|\text{person-years}_{\boldsymbol{a,j}}\boldsymbol{)=}\boldsymbol{B}_{\boldsymbol{0,j}}\boldsymbol{+}\boldsymbol{B}_{\boldsymbol{a,j}}\boldsymbol{+}\text{log(}\text{person-years}_{\boldsymbol{a,j}}\boldsymbol{)}$$

$$\boldsymbol{B}_{\boldsymbol{0,j}}\boldsymbol{=}\boldsymbol{\beta}_{\boldsymbol{0}}\boldsymbol{+\gamma}\boldsymbol{X}_{\boldsymbol{j}}\boldsymbol{+}\boldsymbol{\alpha}_{\boldsymbol{0,j}}$$

$$\boldsymbol{B}_{\boldsymbol{a,j}}\boldsymbol{=}\boldsymbol{\beta}_{\boldsymbol{a}}\boldsymbol{+}\boldsymbol{\eta}_{\boldsymbol{a}}\boldsymbol{X}_{\boldsymbol{j}}\boldsymbol{+}\boldsymbol{\alpha}_{\boldsymbol{a,j}}$$

where ***B_a,j_*** = 0 for 5-15 year olds and $\boldsymbol{\alpha}_{\boldsymbol{0,j}}\boldsymbol{\sim}\text{MVN}\boldsymbol{(0, \Sigma)}$ with $\boldsymbol{\Sigma}$ as the covariance structure of the random effect terms. The linear prediction model is then:

Incidence in children <2 years of age:

$$\log\left( \boldsymbol{\lambda}_{\boldsymbol{1, j}} \right|\text{person-years}_{\boldsymbol{1,j}}\boldsymbol{)}\boldsymbol{=}\boldsymbol{\beta}_{\boldsymbol{0}}\boldsymbol{+}\boldsymbol{\alpha}_{\boldsymbol{0,j}}\boldsymbol{+}\boldsymbol{\beta}_{\boldsymbol{1}}\boldsymbol{+}\boldsymbol{\alpha}_{\boldsymbol{1,j}}\boldsymbol{+\gamma}\boldsymbol{X}_{\boldsymbol{j}}\boldsymbol{+}\boldsymbol{\eta}_{\boldsymbol{1}} \boldsymbol{X}_{\boldsymbol{j}}\boldsymbol{+}\log\left( \text{person-years}_{\boldsymbol{1,j}} \right)$$

Incidence in 2-4 years of age:

$$\log\left( \boldsymbol{\lambda}_{\boldsymbol{2, j}} \right|\text{person-years}_{\boldsymbol{2,j}}\boldsymbol{)}\boldsymbol{=}\boldsymbol{\beta}_{\boldsymbol{0}}\boldsymbol{+}\boldsymbol{\alpha}_{\boldsymbol{0,j}}\boldsymbol{+}\boldsymbol{\beta}_{\boldsymbol{2}}\boldsymbol{+\alpha}_{\boldsymbol{2, j}}\boldsymbol{+\gamma}\boldsymbol{X}_{\boldsymbol{j}}\boldsymbol{+}\boldsymbol{\eta}_{\boldsymbol{2}} \boldsymbol{X}_{\boldsymbol{j}}\boldsymbol{+}\log\left( \text{person-years}_{\boldsymbol{2,j}} \right)$$

Incidence in 5-14 years of age:

$$\log\left( \boldsymbol{\lambda}_{\boldsymbol{3, j}} \right|\text{person-years}_{\boldsymbol{3,j}}\boldsymbol{)=}\boldsymbol{\beta}_{\boldsymbol{0}}\boldsymbol{+}\boldsymbol{\alpha}_{\boldsymbol{0,j}}\boldsymbol{+\gamma}\boldsymbol{X}_{\boldsymbol{j}}\boldsymbol{+}\log\left( \text{person-years}_{\boldsymbol{3,j}} \right)$$

Incidence in **≥**15 years of age:

$$\log\left( \boldsymbol{\lambda}_{\boldsymbol{4, j}} \right|\text{person-years}_{\boldsymbol{4,j}}\boldsymbol{)}\boldsymbol{=}\boldsymbol{\beta}_{\boldsymbol{0}}\boldsymbol{+}\boldsymbol{\alpha}_{\boldsymbol{0,j}}\boldsymbol{+}\boldsymbol{\beta}_{\boldsymbol{4}}\boldsymbol{+ \alpha}_{\boldsymbol{4, j}}\boldsymbol{+\gamma}\boldsymbol{X}_{\boldsymbol{j}}\boldsymbol{+}\boldsymbol{\eta}_{\boldsymbol{4}} \boldsymbol{X}_{\boldsymbol{j}}\boldsymbol{+}\log\left( \text{person-years}_{\boldsymbol{4,j}} \right)$$

where ***γ*** and ***η_a_*** are the effect sizes corresponding to each predictor for the intercept and slope, respectively; again, ***B_a,j_* = 0** for 5-14 year olds and $\boldsymbol{\alpha}_{\boldsymbol{0,j}}\boldsymbol{\sim}\text{MVN}\boldsymbol{(0, \Sigma)}$ where $\boldsymbol{\Sigma}$ has a vague prior distribution described by an inverse Wishart distribution parameterized by a 4x4 identity matrix and 5 degrees of freedom.

A number of studies did not report incidence according to age categories that align with the categories in our model. However, incidence of any combination of the above categories may be modeled as the sum of the rates of the respective age categories. This follows from the fact that the observed cases of a combination of age categories are assumed to result from the combination of Poisson processes, which is equivalent to a Poisson process with a rate parameter equal to the sum of the respective rate parameters, e.g.:

$$\text{Incidence in children of ages <5:} \boldsymbol{\lambda}_{\boldsymbol{1,j}}\boldsymbol{|}\text{person-years}_{\boldsymbol{1,j}}\boldsymbol{+}\boldsymbol{\lambda}_{\boldsymbol{2,j}}\boldsymbol{|}\text{person-years}_{\boldsymbol{2,j}}$$

## ***Model of the Observation Process***

The typhoid surveillance studies featured in our analysis did not follow a standard protocol; therefore, we adjusted for the type of surveillance that was conducted in each of the studies, the participation rate, and the amount of blood that was collected to culture S. typhi (Figure 1).

***Surveillance***. We identified three types of studies. Within population-based studies, there were active (or “augmented passive”) surveillance, in which passive surveillance at a health center was augmented by weekly or biweekly visits to households to ask about febrile illness and encourage study participation; and there were passive surveillance studies, in which the catchment population was enumerated, and surveillance was only done at one or more health centers serving that population. A third type of study was identified as hospital-based passive surveillance, in which the population under surveillance was not enumerated, but there was an indication that most cases diagnosed at the study health center(s) came from the local catchment population and that most cases in that population would attend the study health center(s). Preliminary analysis showed that there were insufficient data to estimate a difference in case ascertainment between passive population-based and hospital-based surveillance, and therefore we estimated only one parameter to adjust for the differences observed between passive or hospital-based surveillance versus active or augmented passive surveillance studies.

$$\text{Cases detected by surveillance}\boldsymbol{\sim}\text{Binomial}\boldsymbol{(}\text{Actual Cases}\boldsymbol{,}\boldsymbol{\phi}_{\boldsymbol{s}}\boldsymbol{)}$$

$$\boldsymbol{\phi}_{\boldsymbol{s}}\boldsymbol{=}\left\{ \begin{matrix} \text{\textasciitilde Beta}\left( \boldsymbol{1,1} \right)\text{ for passive surveillance} \\ \boldsymbol{=1}\text{ for active surveillance} \end{matrix} \right.$$

We estimated a single parameter ***ϕ_s_*** representing the average effect of passive surveillance (as opposed to active surveillance) across all age groups; there was not enough data to estimate age-specific differences.

***Participation Rate***. In addition, due to operational constraints or the source populations’ willingness to participate in clinical studies, most surveillance studies reported a “participation rate” among the source population. For example, it may not have been possible to perform blood cultures for all children presenting with prolonged fever in malaria-endemic study sites [11] and therefore we modeled the probability that a true typhoid case would participate in the study (i.e. attend a study hospital and have blood drawn for diagnosis) as an additional binomial process:

$$\text{Cases that agreed to participate}\boldsymbol{\sim}\text{Binomial}\boldsymbol{(}\text{Cases detected by surveillance}\boldsymbol{,}\boldsymbol{\phi}_{\boldsymbol{p,j,a}}\boldsymbol{)}$$

The “participation rate” ***φ_j,a_*** was set equal to the reported (age-specific) participation rate in study site *j*. Studies that did not report a participation rate were assumed to have observed 100% participation.

***Blood Culture Sensitivity.*** Finally, observed cases are influenced by one more binomial process related to the test sensitivity of blood culture (the standard diagnostic), which is recognized to be suboptimal (i.e. only 40-60%)[12,13] Adjusting for blood culture sensitivity is important in order to avoid underestimating the true burden of the disease. Moreover, since less blood is typically drawn from younger children, it is important to understand how age-related differences in incidence may be attributable to differences in culture sensitivity by age. Thus, we assumed the number of observed (culture-confirmed) typhoid cases in age group ***a*** in each surveillance site ***j*** represented a fraction $\boldsymbol{\phi}_{\boldsymbol{c,j,a}}$of the estimated number of cases that would have been detected using a perfect (100% sensitive and specific) test:

$$\text{Observed blood-culture positive cases}\boldsymbol{\sim}\text{Binomial}\left( \text{Cases that agreed to participate}\boldsymbol{,}\boldsymbol{\phi}_{\boldsymbol{c,j,a}} \right)$$

We surveyed the diagnostic accuracy literature on blood culture sensitivity of typhoid cases confirmed by the bone marrow culture [14–21]. Because the sensitivity of blood culture depends on the amount of blood drawn from the patient, a meta-regression allowed us to estimate the relationship between amount of blood drawn and culture sensitivity, which we used to parameterize the prior distribution for $\boldsymbol{\phi}_{\boldsymbol{c,j,a}}$ in our incidence model. The functional form of this parameter aims to integrate two distinct types of studies: one set of studies measured the test sensitivity of patients using a fixed volume of blood, and a second set of studies measured the test sensitivity of patients using two different amounts of blood on the same patients, which allowed us to understand whether an additional mL of blood would render the blood culture of a typhoid positive. Therefore, we chose probability function analogous to survival functions in order to take into account studies with multiple measures. Samples where no *S.* Typhi grew in the blood culture were treated as right-censored observations—either the amount of blood collected or more would be necessary for the blood culture to be positive.

Patients whose blood culture was positive at volume $\nu_{1}:$

$$\boldsymbol{\pi}_{\boldsymbol{1}}\boldsymbol{=}\mathbf{1-}\exp\left( \mathbf{-}\boldsymbol{\psi}\text{ × volume}_{\boldsymbol{1}} \right)$$

Patients whose blood culture was negative at volume $\nu_{1}$ but positive at volume $\nu_{2}$:

$$\boldsymbol{\pi}_{\boldsymbol{2}}\mathbf{=[1- exp}\left( \mathbf{-}\boldsymbol{\psi}\text{ × volume}_{\boldsymbol{2}} \right)\mathbf{]- [1-exp}\left( \mathbf{-}\boldsymbol{\psi}\text{ × volume}_{\boldsymbol{1}} \right)\mathbf{]}$$

Patients whose blood culture was negative at volume $\nu_{2}$:

$$\boldsymbol{\pi}_{\boldsymbol{3}}\boldsymbol{=}\mathbf{exp(-}\boldsymbol{\psi}\text{ × volume}_{\boldsymbol{2}}\mathbf{)}$$

The likelihood function for each observation is:

$$\boldsymbol{Observation \sim Multinomial(\pi, All typhoid cases in the study)}$$

Lastly, because unmeasured heterogeneity between studies may impact the observations, we fit the $\boldsymbol{\psi}$parameter as a random effect, which allowed us to be more conservative in our conclusions on the effect of blood sample volume and sensitivity. The random effects were distributed as a log-normal distribution (since the rate parameter must be positive) and two hyper-parameters were estimated simultaneously to determine the common distribution of all random effects.

$$\boldsymbol{\psi}_{\boldsymbol{j}}\boldsymbol{\sim}\mathbf{Log}\text{Normal}\left( \boldsymbol{\mu, \sigma} \right)$$

Predictions of sensitivity were made by simulating the ***π*** as a function of the random effect hyper-parameter, ***μ***, as shown in Figure S1. It is notable that below 2 mL of blood, we cannot make any inference on the sensitivity of the blood culture. Moreover, there is a degree of “diminishing returns” in terms of the gains in sensitivity that are acquired as one collects increasingly more blood. In our main analysis, we used the exponentiated values of posterior draws of $\boldsymbol{\mu}$ to address the inherent correlation between the sensitivity of the two volumes.

If an incidence study did not report the blood culture protocol, we assumed all children 0-4 years old had 4 mL of blood drawn, and all children 5 years old or older had 7 mL of blood drawn, as per a review of the study protocols in the Diseases of the Most Impoverished (DOMI) studies, which constituted eight out of the 28 typhoid incidence studies in this analysis[22].

Integrating the observation processes and the disease process into our estimation algorithm, we obtained the following relationship:

$$\text{Observed Cases}\boldsymbol{|}\text{Population}\boldsymbol{\sim}\text{Poisson}\boldsymbol{(}\boldsymbol{\lambda}_{\boldsymbol{a,j}}\boldsymbol{\phi}_{\boldsymbol{s}}\boldsymbol{\phi}_{\boldsymbol{p,j,a}}\boldsymbol{\phi}_{\boldsymbol{c,j,a}}\boldsymbol{)}$$

# **Variable selection and model estimation**

Prediction models such as ours should incorporate the uncertainty not just in model parameters but also in model composition. Including all of the predictors may yield a model with poor predictive power. In other words, our predictions should be derived from a suite of models that represent different combinations of predictors, weighted by the likelihood of each model. Model averaging techniques would require estimating all possible models, equal to all of the combinations of predictors, ***p***: this would constitute **3*^p^*** models if the predictors are included in the intercept (***B*_0,_*_j_***) as well as the slopes (***B_a_*_,_*_j_***). Adequately estimating each model via Markov Chain Monte Carlo (MCMC) and assessing convergence would be computationally expensive, and sensitivity analyses would be intractable. However, a MCMC algorithm using spike-and-slab priors allowed us to estimate the parameters of the model as well as search through the model space for the predictor combinations that best describe the data.

Spike-and-slab priors have been described elsewhere[23,24], but briefly, this method consists of providing two latent priors for the coefficient for each predictor; one latent prior is (typically) a Bernoulli draw that determines whether the coefficient for a given prediction is set to zero or whether the coefficient takes the value of the second latent prior, a continuous parameter normally distributed with large prior variance. We replaced the typical Bernoulli-distributed latent variable with a categorically-distributed variable that would determine if a predictor was 1) excluded from the model, 2) included in the model as a predictor of the intercept (***B*_0,_*_j_***) only, or 3) entered the model as a predictor of the intercept as well as the slopes (***B_a_*_,_*_j_***). We then estimated the model using a Metropolis-Hastings algorithm that not only samples from the posterior distribution of the model parameters, but that also samples from the model space via a random walk with a “step-size” equal to one more or one fewer predictor in the model. Specific details about how we employed spike-and-slab priors in our analyses are below.

## ***Characterizing the model space***

Ordinarily, the number of models that may include any ***m*** predictors of a total ***p*** predictors is $\sum_{\boldsymbol{m=0}}^{\boldsymbol{p}} \binom{\boldsymbol{p}}{\boldsymbol{m}}$= **2*^p^*.** The model space expands considerably if one then tests any combination of the interaction between a variable other than one of the ***p*** predictors and any of the ***m*** selected main effects. Note that one must include the predictor as a fixed effect of the intercept if one wants to include it as well as a fixed effect of the slope.

Therefore, the number of models that would be tested if we were to estimate all the models combinations described in this paper would be **3*^p^***:

$$\sum_{\boldsymbol{m=0}}^{\boldsymbol{p}} \binom{\boldsymbol{p}}{\boldsymbol{m}}\sum_{\boldsymbol{n=0}}^{\boldsymbol{m}} \binom{\boldsymbol{m}}{\boldsymbol{n}}\boldsymbol{=}\sum_{\boldsymbol{m=0}}^{\boldsymbol{p}} \binom{\boldsymbol{p}}{\boldsymbol{m}}\boldsymbol{2}^{\boldsymbol{m}}\boldsymbol{=}\boldsymbol{3}^{\boldsymbol{p}}$$

Which is the solution to the ordinary generating function:

$$\sum_{\boldsymbol{k}}^{\boldsymbol{n}} \binom{\boldsymbol{n}}{\boldsymbol{k}}\boldsymbol{x}^{\boldsymbol{k}}\boldsymbol{=}\boldsymbol{(1+x)}^{\boldsymbol{n}}$$

For any one predictor one may choose to include in the model, either as a predictor of the intercept or of the slope, a third of the models do not contain the predictor at all, a third of the models contain the predictor as a fixed effect of the intercept, and a third of the models contain that predictor as a fixed effect of both the intercept and the slopes.

## ***Variable selection for fixed effects via spike and slab priors***

We identified 12 predictors to consider in our models, and employed spike-and-slab priors on the coefficients of these predictors to explore the space of all possible models and simultaneously estimate parameters via MCMC using a Metropolis Hastings sampler. Variables under selection have a diffuse prior, or a "slab"; the "spike" refers to the probability of excluding that predictor, or assigning a value of zero. A standard spike-and-slab prior contains two components:

$$\boldsymbol{\Gamma\sim}\text{Bernoulli}\left( \boldsymbol{0.5} \right)$$

$$\boldsymbol{\gamma}_{\text{prior}}\boldsymbol{\sim}\text{Normal}\boldsymbol{(0, 1000)}$$

$$\boldsymbol{\gamma=\Gamma}\boldsymbol{\gamma}_{\text{prior}}$$

As long as a predictor remains out of the model, the posterior for $\boldsymbol{\gamma}_{\text{prior}}$ (the "slab") spans the prior distribution. When it is selected to be in the model, the posterior takes on a narrow range around the estimate of the coefficient. It is important for the "slab" portion of the coefficient have an uninformative distribution that can allow the data to determine the distribution of the posteriors.

Since our models include predictors for the intercept as well as for the slope of the age coefficient, we had to modify the scheme above so that each predictor could enter two-thirds of the models; in one-third of the models the predictor is used to estimate the intercept, and in one-third of the models the predictor is used to estimate the intercept and slopes of the model. We therefore replaced the prior on the selection parameter **Γ** with a categorical distribution with three parameters:

$$\boldsymbol{\Gamma\sim Categorical}\left( \frac{\boldsymbol{1}}{\boldsymbol{3}}\boldsymbol{,}\frac{\boldsymbol{1}}{\boldsymbol{3}}\boldsymbol{,}\frac{\boldsymbol{1}}{\boldsymbol{3}} \right)$$

$$\left\langle\boldsymbol{\gamma, \eta} \right\rangle\boldsymbol{=}\left\{ \begin{aligned} \left\langle\boldsymbol{0,0,0,0} \right\rangle\boldsymbol{, & \Gamma=1} \\ \left\langle\boldsymbol{\gamma}_{\text{prior}}^{\text{intercept}}\boldsymbol{, 0, 0,0} \right\rangle\boldsymbol{, & \Gamma=2} \\ \left\langle{\boldsymbol{\gamma}_{\text{prior}}^{\text{intercept}}\boldsymbol{, \eta}}_{\text{prior}}^{\text{slope}\boldsymbol{1}}\boldsymbol{,}\boldsymbol{\eta}_{\text{prior}}^{\text{slope}\boldsymbol{2}}\boldsymbol{,}\boldsymbol{\eta}_{\text{prior}}^{\text{slope}\boldsymbol{3}} \right\rangle\boldsymbol{, & \Gamma=3} \end{aligned} \right.$$

Ordinarily, the coefficient for each predictor value is given an uninformative prior of Normal(0,1000). However, since we had 12 predictors for selection in the model, the stochastic search through such an expansive parameter space would be computationally unfeasible, therefore we modified the prior distribution (uniform distribution bounded by τ) to accelerate chain mixing:

$$\boldsymbol{\gamma}_{\text{prior}}^{\text{intercept}}\boldsymbol{\sim}\text{Uniform}\boldsymbol{(-\tau, \tau)}$$

$$\boldsymbol{\eta}_{\text{prior}}^{\text{slope}\boldsymbol{a}}\boldsymbol{\sim}\text{Uniform}\boldsymbol{(}\boldsymbol{-\tau}_{\boldsymbol{a}}\boldsymbol{,}\boldsymbol{\tau}_{\boldsymbol{a}}\boldsymbol{)}$$

$\boldsymbol{\tau\sim}\text{Uniform}\boldsymbol{(1,5)}$ and $\boldsymbol{\tau}_{\boldsymbol{a}}\boldsymbol{\sim}\text{Uniform}\boldsymbol{(1,5)}$

We checked that the choice of upper and lower bounds of the hyper-parameters, ***τ*** and ***τ_a_***, would not hinder the estimates of the posterior distributions by limiting the parameter space allowed by the prior distribution for the posterior search (Figure S2).

## ***Computational Tools***

The model was fit using the JAGS (Just Another Gibbs Sampler) software, version 3.4.0, in conjunction with MATLAB 2014b via the MATLAB interface for JAGS, MATJAGS (graciously provided by Mark Steyvers at the University of California, Irvine)[25,26]. We used MATLAB's Parallel Computing Toolbox to call multiple JAGS sessions that would estimate the model with alternative assumptions.

With the exception of the priors for the diagnostic test sensitivity, we provided uninformative or vague priors for all parameters, and all covariates were normalized in order to facilitate mixing of the Markov chains. We drew 10 million samples from the posterior distribution of all parameter estimates after discarding the initial 100,000 burn in iterations, and applied a thinning parameter equal to 10 in order to curtail the degree of auto-correlation among consecutive samples within chains. For validation, where we left 3 locations out of the analysis each time we resampled the model, we re-estimated parameters by drawing 5 million samples and applying a thinning parameter equal to 10. After examining the rate of convergence of our initial estimation model, we determined that we could resample the model by drawing 5 million samples (rather than 20 million samples) in order to get similar results as a longer chain.

To assess convergence on the space of predictor combinations, we ran two Markov chains, one initiated with a null model and one initiated with a saturated model; if each covariate was selected for inclusion with approximately equal chance in both chains, then we concluded that the algorithm had converged. We visually inspected trace plots to assess convergence on the parameter space.

## ***Predictive intervals of incidence rate***

We sampled the posterior parameter combinations in order to estimate the 95% credible intervals of predictive incidence rates. Our predictive model included only the fixed effects for the mean response, since the random effect for a location with no previous population-based incidence study is unknown. We capped incidence at 10,000 per 100,000 persons because that was ten time the highest observed incidence in Delhi, India in 1996.[27]

The model was able to capture some within-country heterogeneity in incidence attributable to the subnational-level predictions. Therefore, our posterior model was calculated on a 0.1 by 0.1-degree lattice. We calculated cases per grid cell by multiplying the incidence by the population density and multiplying it by the area of the cell (as determined by a spherical model of the earth). Region-level case estimates were calculated by integrating the incidence over the raster area representing the region.

Maps of the posterior prediction of incidence were created using MATLAB’s Mapping Toolbox.

**References**1. Crump JA, Luby SP, Mintz ED. The global burden of typhoid fever. Bull World Health Organ. 2004;82: 346–53.

2. Crump JA, Ram PK, Gupta SK, Miller MA, Mintz ED. Part I. Analysis of data gaps pertaining to Salmonella enterica serotype Typhi infections in low and medium human development index countries, 1984-2005. Epidemiol Infect. 2008;136: 436–48. doi:10.1017/S0950268807009338

3. Buckle GC, Walker CLF, Black RE. Typhoid fever and paratyphoid fever: Systematic review to estimate global morbidity and mortality for 2010. J Glob Health. 2012;2: 10401. doi:10.7189/jogh.02.010401

4. Mogasale V, Maskery B, Ochiai RL, Lee JS, Mogasale V V, Ramani E, et al. Burden of typhoid fever in low-income and middle-income countries: a systematic, literature-based update with risk-factor adjustment. Lancet Glob Heal. Mogasale et al. Open Access article distributed under the terms of CC BY-NC-SA; 2014;2: e570-80. doi:10.1016/S2214-109X(14)70301-8

5. World Bank. World Development Indicators. Washington, DC.: World Bank (producer and distributor); 2015.

6. Gridded Population of the World, Version 3 (GPWv3): National Identifier Grid. Palisades, NY: NASA Socioeconomic Data and Applications Center (SEDAC); 2005.

7. Nordhaus WD. Geography and macroeconomics: new data and new findings. Proc Natl Acad Sci U S A. 2006;103: 3510–7. doi:10.1073/pnas.0509842103

8. Nordhaus WD, Chen X. Geography: Graphics and Economics. B E J Econ Anal Policy. 2009;9.

9. Adhikari P, Hong Y, Douglas KR, Kirschbaum DB, Gourley J, Adler R, et al. A digitized global flood inventory (1998-2008): Compilation and preliminary results. Nat Hazards. 2010;55: 405–422. doi:10.1007/s11069-010-9537-2

10. Mekonnen MM, Hoekstra AY. Four billion people facing severe water scarcity. Sci Adv. 2016;2: e1500323. doi:10.1126/sciadv.1500323

11. Breiman RF, Cosmas L, Njuguna H, Audi A, Olack B, Ochieng JB, et al. Population-based incidence of typhoid fever in an urban informal settlement and a rural area in Kenya: implications for typhoid vaccine use in Africa. PLoS One. United States; 2012;7: e29119. doi:10.1371/journal.pone.0029119

12. Wain J, Hosoglu S. The laboratory diagnosis of enteric fever. J Infect Dev Ctries. 2008;2: 421–5.

13. Wain J, Diep TS, Bay PVB, Walsh AL, Vinh H, Duong NM, et al. Specimens and culture media for the laboratory diagnosis of typhoid fever. J Infect Dev Ctries. 2008;2: 469–74. doi:http://dx.doi.org/10.3855/jidc.164

14. Parry CM, Wijedoru L, Arjyal A, Baker S. The utility of diagnostic tests for enteric fever in endemic locations. Expert Rev Anti Infect Ther. 2011;9: 711–25. doi:10.1586/eri.11.47

15. Darton TC, Blohmke CJ, Pollard AJ. Typhoid epidemiology, diagnostics and the human challenge model. Curr Opin Gastroenterol. 2014;30: 7–17. doi:10.1097/MOG.0000000000000021

16. Gilman RH, Terminel M, Levine MM, Hernandez-Mendoza P, Hornick RB. Relative efficacy of blood, urine, rectal swab, bone-marrow, and rose-spot cultures for recovery of Salmonella typhi in typhoid fever. Lancet. 1975;1: 1211–3.

17. Guerra-Caceres JG, Gotuzzo-Herencia E, Crosby-Dagnino E, Miro-Quesada M, Carrillo-Parodi C. Diagnostic-Value of Bone-Marrow Culture in Typhoid-Fever. Trans R Soc Trop Med Hyg. 1979;73: 680–683. doi:10.1016/0035-9203(79)90020-8

18. Vallenas C, Hernandez H, Kay B, Black R, Gotuzzo E. Efficacy of bone marrow, blood, stool and duodenal contents cultures for bacteriologic confirmation of typhoid fever in children. Pediatr Infect Dis J. 1985;4: 496–8.

19. Hoffman SL, Edman DC, Punjabi NH, Lesmana M, Cholid A, Sundah S, et al. Bone marrow aspirate culture superior to streptokinase clot culture and 8 ml 1:10 blood-to-broth ratio blood culture for diagnosis of typhoid fever. Am J Trop Med Hyg. 1986;35: 836–9.

20. Gasem MH, Dolmans WM, Isbandrio BB, Wahyono H, Keuter M, Djokomoeljanto R, et al. Culture of Salmonella typhi and Salmonella paratyphi from blood and bone marrow in suspected typhoid fever. Trop Geogr Med. Amsterdam, Netherlands; 1995;47: 164–167.

21. Gasem MH, Smits HL, Goris MGA, Dolmans WM V. Evaluation of a simple and rapid dipstick assay for the diagnosis of typhoid fever in Indonesia. J Med Microbiol. 2002;51: 173–177.

22. Ochiai RL, Acosta CJ, Danovaro-Holliday MC, Baiqing D, Bhattacharya SK, Agtini MD, et al. A study of typhoid fever in five Asian countries: Disease burden and implications for controls. Bull World Health Organ. 2008;86: 260–268. doi:10.2471/BLT.06.039818

23. George EI, McCulloch RE. Variable Selection via Gibbs Sampling. Journal of the American Statistical Association. 1993. pp. 881–889. doi:10.1080/01621459.1993.10476353

24. Ishwaran H, Rao JS. Spike and slab variable selection: Frequentist and bayesian strategies. Ann Stat. 2005;33: 730–773. doi:10.1214/009053604000001147

25. Plummer M. JAGS: A program for analysis of Bayesian graphical models using Gibbs sampling. Proc 3rd Int Work Distrib Stat Comput. 2003; 1–10. doi:ISSN 1609-395X

26. Steyvers M, Kalish M. MATJAGS, a Matlab interface for JAGS. 2014.

27. Sinha A, Sazawal S, Kumar R, Sood S. Typhoid fever in children aged less than 5 years. Lancet. 1999;354: 734–737.
