# Supplementary material for: The burden of typhoid fever in low- and middle-income countries: A meta-regression approach
Source: PLoS Negl Trop Dis. 2017 Feb 27;11(2):e0005376. doi: 10.1371/journal.pntd.0005376 (PMC5344533; doi:10.1371/journal.pntd.0005376)
Supplement: S1 Table — Incidence rates (and 95% confidence intervals) per 100,000 person-years. Incidence rates shown are not adjusted for participation rate, surveillance type, or blood culture sensitivity. (DOCX) [file pntd.0005376.s002.docx]

**Table S1.** **Incidence studies used to estimate the parameters of the prediction model.** Incidence rates (and 95% confidence intervals) per 100,000 person-years. Incidence rates shown are not adjusted for participation rate, surveillance type, or blood culture sensitivity.

| **Continent** | **Country (ISO3)** | **Location of study** | **Type of surveillance*** | **<2 years old** | **2-4 year olds** | **5-15 year olds** | ≥**15 year olds** | **Study period** | **Source** |
| --- | --- | --- | --- | --- | --- | --- | --- | --- | --- |
| Africa | Kenya | Kibera, Nairobi, Kenya | Active or augmented passive surveillance | 67 (2, 248) | 226 (91, 421) | 401 (295, 522) | 89 (63, 119) | March 2007 – March 2009 | Breiman (2012) [11] |
|  |  | Lwak, Rural West Kenya | Active or augmented passive surveillance | 15 (0, 56) | 28 (3, 79) | 21 (8, 42) | 37 (20, 59) | March 2007 – March 2009 | Breiman (2012) [11] |
|  | South Africa | Transvaal, South Africa | Active or augmented passive surveillance |  |  | 494 (423, 570) |  | November 1985 - August 1987 | Klugman (1987) [22] |
|  | Ghana | Ashanti, Ghana | Passive surveillance | 38 (1, 138) | 200 (86, 361) | 58 (23, 109) |  | September 2007 - November 2008 | Marks (2010) [23] |
|  | Egypt | Bilbeis District, Egypt | Passive surveillance | 0 (0, 28) | 0 (0, 28) | 10 (6, 15) | | August 2000 - January 2001 | Crump (2003) [24] |
|  |  | Fayoum Governorate, Egypt | Passive surveillance |  | 1 (0, 4) | 25 (19, 32) | 6 (4, 8) | June 9 - October 31, 2002 | Srikantiah (2006) [25] |
|  |  | Alexandria, Egypt | Passive surveillance |  |  | 46 (29, 67) |  | March 1978 – March 1981 | Wahdan (1982) [26] |
| Asia | Pakistan | Karachi, Pakistan | Active or augmented passive surveillance | 0 (0, 95) | 111 (59, 180) | 264 (185, 357) |  | June 1999 – December 2001 | Siddiqui (2006) [27] |
|  |  | Hijrat, Sultanabad, and Bilal colonies in Karachi, Pakistan | Active or augmented passive surveillance |  | 230 (122, 371) | 190 (133, 257) |  | January 2002 – December 2007 | Khan (2012) [28] |
|  |  | Sultanabad and Hijrat, Karachi, Pakistan | Active or augmented passive surveillance |  | 573 (435, 730) | 413 (345, 487) |  | May 2003 – April 2004 | Ochai 2008 [16] |
|  |  | Rehri Goth | Active or augmented passive surveillance | 443 (178, 827) | 380 (174, 665) |  |  | February 2007 – May 2008 | Owais (2010) [29] |
|  | Bangladesh | Kalamapur, Dhaka, Bangladesh | Active or augmented passive surveillance | 630 (130, 1517) | 1150 (720, 1679) | 87 (48, 136) | | January 2003 – January 2004 | Naheed (2010) [30] |
|  |  |  | Active or augmented passive surveillance | 1391 (601, 2508) | 2203 (1306, 3332) | 524 (279, 845) | 117 (56, 200) | December 2000 – October 2001 | Abdullah Brooks (2005) [31] |
|  | India | Kalkaji, Dehli, India | Active or augmented passive surveillance | 1359 (441, 2783) | 3490 (2212, 5054) | 1167 (798, 1604) | 112 (23, 269) | November 1995 – October 1996 | Sinha (1999) [21] |
|  |  |  | Passive surveillance | 250 (174, 339) | | | | November 1995-February 1997 | Bahl 2004 [32] |
|  |  | Wards 29 and 30 in Kolkata, India | Active or augmented passive surveillance | 67 (2, 248) | 226 (91, 421) | 401 (295, 522) | 89 (63, 119) | January 2004 – December 2004 | Sur (2006) [33] |
|  |  |  | Active or augmented passive surveillance |  | 1233 (812, 1740) | 589 (442, 756) | 57 (32, 89) | November 2004 – December 2006 | Sur (2009) [34] |
|  | Nepal | Villages adjacent to Kathmandu, Nepal | Active or augmented passive surveillance | 928 (634, 1275) | | | | April 1986 - July 1987 | Acharya (1987) [35] |
|  | Indonesia | Plaju and Sumai Gerong, Sumatra, Indonesia | Passive surveillance |  | 1307 (976, 1685) | 1230 (1011, 1471) | 361 (264, 472) | October 1986 - March 1989 | Simanjuntak (1990) [36] |
|  |  | North Jakarta, Indonesia | Passive surveillance | 7 (0, 27) | 122 (76, 178) | 135 (113, 159) | 36 (28, 45) | August 2001 - July 2003 | Punjabi (2013) [37] |
|  | China | Quan, Guangxi Zhuang, China | Passive surveillance |  | 0 (0, 1194) | 33 (19, 51) | 41 (15, 80) | April 1995 – November 1996 | Yang (2001) [38] |
|  |  | Hechi/Quan, Guangxi Zhuang, China | Passive surveillance |  |  | 29 (10, 60) | 12 (6, 21) | August 2001 – July 2002 | Ochiai (2008) [16] |
|  | Vietnam | MH, MT, MX communes in Dong Thap Province, Vietnam | Passive surveillance | 0 (0, 310) | 336 (123, 654) | 474 (332, 641) | 110 (60, 175) | December 1995 – December 1996 | Lin (2000) [39] |
|  |  |  | Active or augmented passive surveillance |  | 548 (378, 751) |  |  | March 1998 – Feb 1999 | Lin (2001) [40] |
|  |  |  | Active or augmented passive surveillance |  | 306 (194, 443) |  |  | March 1999 – May 2000 | Lin (2001) [40] |
|  |  |  | Passive surveillance |  |  | 212 (123, 323) |  | May 2000 – December 2001 | Lanh (2004)[41] |
|  |  | Hue, Vietnam | Passive surveillance |  |  | 24 (14, 37) | 11 (1, 30) | June 2002 – June 2003** | Ochiai (2008) [16] |
|  | Uzbekistan | Samarkand City, Uzbekistan | Passive surveillance | 0 (0, 30) | 0 (0, 20) | 54 (40, 70) | 28 (21, 36) | July 2002 – May 2003 | Srikantiah (2007) [42] |
| Latin America & Caribbean | Chile | Santiago, Chile | Passive surveillance | 227 (174, 287) | | | | July 1, 1982 - June 20, 1987 | Black (1990) [43] |
|  |  | Santiago, Chile (Area Occidente) | Passive surveillance |  |  | 112 (85, 143) | 87 (46, 140) | September 1983 – September 1986 | Levine (1987) [44] |
|  |  | Santiago, Chile (Area Sur Oriente & Area Norte) | Passive surveillance |  |  | 91 (60, 127) |  | November 1986 – November 1989 | Levine (1990) [45] |
|  | Haiti | Artibonite Valley, Haiti | Passive surveillance | 112 (102, 124) | | | 80 (70, 91) | January 1988 - July 1991 | Olle-Goig (1993) [46] |

*Active or augmented passive surveillance entailed surveillance designs that included periodic home visits to inquire about the health status of household members. Blood culture was always contingent on attending a healthcare facility.

** We used the estimates of cases and population from Ochai 2008 instead of Khan 2006 because Ochai 2008 included data from Khan 2006 as well as surveillance in a third colony of Karachi.

† Sources correspond to references in Text S1.
