# Supplementary material for: The burden of typhoid fever in low- and middle-income countries: A meta-regression approach
Source: PLoS Negl Trop Dis. 2017 Feb 27;11(2):e0005376. doi: 10.1371/journal.pntd.0005376 (PMC5344533; doi:10.1371/journal.pntd.0005376)
Supplement: S2 Table — Incidence rates (and 95% confidence intervals) per 100,000 person-years. Incidence rates shown are not adjusted for participation rate, surveillance type, or blood culture sensitivity. (DOCX) [file pntd.0005376.s003.docx]

**Table S2. Incidence studies used to validate our model.** Incidence rates (and 95% confidence intervals) per 100,000 person-years. Incidence rates shown are not adjusted for participation rate, surveillance type, or blood culture sensitivity.

| **Country** | **Location of study** | **Type of surveillance** | **<2 years old** | **2-4 year olds** | **5-15 year olds** | **15+ year olds** | **Study period** |
| --- | --- | --- | --- | --- | --- | --- | --- |
| Burkina Faso | Nioko II | Passive, population-based | 0 (0, 176) | 48 (1, 176) | 82 (22, 179) | 0 (0, 35) | April 2012 - September 2013 |
|  | Polesgo | Passive, population-based | 0 (0, 397) | 605 (222, 1176) | 238 (77, 487) | 41 (5, 113) | April 2012 - September 2014 |
| Guinea Bissau | Bandim | Passive, population-based | 0 (0, 71) | 26 (1, 95) | 9 (0, 33) | 3 (0, 10) | December 2011 - April 2013 |
| Kenya | Kibera | Active or augmented passive surveillance | 148 (30, 356) | 490 (235, 838) | 489 (325, 687) | 141 (75, 227) | January 2012 - December 2013 |
| Madagascar | Imerintsiatosika | Passive, population-based | 0 (0, 287) | 0 (0, 191) | 123 (40, 252) | 14 (0, 52) | November 2011 - June 2013 |
|  | Antananarivo | Passive, population-based | 0 (0, 1060) | 0 (0, 705) | 50 (1, 184) | 36 (4, 99) | February 2012 - May 2013 |
| Tanzania | Moshi – rural | Passive, population-based | 0 (0, 532) | 0 (0, 512) | 95 (26, 209) | 38 (5, 105) | September 2011 - May 2013 |
|  | Moshi – urban | Passive, population-based | 0 (0, 620) | 809 (263, 1657) | 16 (6, 30) | 67 (25, 131) | September 2011 - May 2014 |
| Ghana | Ashanti Akim North | Passive, population-based | 49 (6, 137) | 476 (260, 756) | 127 (73, 197) |  | March 2010 – May 2012 |
