# Supplementary material for: The burden of typhoid fever in low- and middle-income countries: A meta-regression approach
Source: PLoS Negl Trop Dis. 2017 Feb 27;11(2):e0005376. doi: 10.1371/journal.pntd.0005376 (PMC5344533; doi:10.1371/journal.pntd.0005376)
Supplement: S3 Table — (DOCX) [file pntd.0005376.s004.docx]

**Table S3. Random effect variance-covariance matrix.**

|  | ***α_0_*** | ***α_1_*** | ***α_2_*** | ***α_3_*** |
| --- | --- | --- | --- | --- |
| ***α_0_*** | 0.73 |  |  |  |
| ***α_1_*** | 0.42 | 2.37 |  |  |
| ***α_2_*** | 0.28 | 1.12 | 1.14 |  |
| ***α_3_*** | -0.21 | 0.25 | -0.17 | 0.32 |

**α_0 =_** Random effect for the log incidence rate of 5-15 year olds

**α_1 =_** Random effect for the log incidence rate ratio between 0-2 year olds and 5-15 year olds

**α_2 =_** Random effect for the log incidence rate ratio between 2-5 year olds and 5-15 year olds

**α_3 =_** Random effect for the log incidence rate ratio between adults and 5-15 year olds

Note: these are the variance-covariance estimates of incidence on the natural log scale.
