# Supplementary material for: The burden of typhoid fever in low- and middle-income countries: A meta-regression approach
Source: PLoS Negl Trop Dis. 2017 Feb 27;11(2):e0005376. doi: 10.1371/journal.pntd.0005376 (PMC5344533; doi:10.1371/journal.pntd.0005376)
Supplement: S4 Table — We ran a null model assuming regional hyperpriors for the location-specific random effects (in addition to a global hyperprior) using two schemes to group countries: continents and the Global Burden of Disease (GBD) Regions for 2015. We discovered that regional hyperpriors would not differ significantly from global hyperpriors. Further, we note that there are only data for two locations in the Americas, making a separate hyperprior for this region unnecessary. (DOCX) [file pntd.0005376.s005.docx]

**Table S4. Posterior distributions for a model with 2-level hyperpriors to estimate random effects.**

We ran a null model assuming regional hyperpriors for the location-specific random effects (in addition to a global hyperprior) using two schemes to group countries: continents and the Global Burden of Disease (GBD) Regions for 2015. We discovered that regional hyperpriors would not differ significantly from global hyperpriors. Further, we note that there are only data for two locations in the Americas, making a separate hyperprior for this region unnecessary.

|  | **Null Model** | | | | **Model with covariates** | | | |
| --- | --- | --- | --- | --- | --- | --- | --- | --- |
|  | **Incidence of children 5-14 (natural log)** | **IRR for children <2 (natural log)** | **IRR for children 2-4 (natural log)** | **IRR for adults ≥15 (natural log)** | **Incidence of children 5-14 (natural log)** | **IRR for children <2 (natural log)** | **IRR for children 2-4 (natural log)** | **IRR for adults ≥15 (natural log)** |
| Continents | | | | | | | | |
| Global Hyperprior | -5.7 (-6.9, -4.5) | -0.8 (-2.9, 1.4) | 0.8 (-0.8, 2.6) | -1.0 (-2.1, 0.2) | -5.4 (-6.7, -4.2) | -1.3 (-3.3, 0.4) | 0.2 (-1.4, 1.7) | -1.1 (-2.2, 0.1) |
| Africa | -6.1 (-6.9, -5.3) | -0.5 (-2.2, 0.8) | 0.5 (-0.9, 1.9) | -1.1 (-1.9, -0.3) | -5.6 (-6.8, -4.6) | -1.3 (-3.1, 0.2) | -0.2 (-1.4, 1.0) | -1.2 (-2.1, -0.3) |
| America | -5.7 (-7.0, -4.6) | -0.6 (-3.6, 3.0) | 1.6 (-0.8, 3.4) | -0.7 (-1.9, 0.5) | -5.3 (-6.7, -4.0) | -1.3 (-3.7, 1.0) | 0.3 (-1.9, 2.2) | -0.9 (-2.0, 0.3) |
| Asia | -5.3 (-6.0, -4.6) | -1.2 (-2.8, 0.2) | 0.5 (-0.3, 1.2) | -1.3 (-1.7, -0.8) | -5.4 (-6.1, -4.6) | -1.3 (-3.0, 0.0) | 0.5 (-0.7, 1.5) | -1.2 (-1.8, -0.7) |
| Global Burden of Disease (GBD) Regions | | | | | | | | |
| Global Hyperprior | -5.9 (-6.9, -5.0) | -1.6 (-4.7, 0.4) | -0.1 (-2.3, 1.8) | -1.1 (-1.9, -0.4) | -5.5 (-6.3, -4.6) | -1.3 (-3.5, 0.1) | 0.2 (-1.5, 1.4) | -1.1 (-1.9, -0.3) |
| Eastern Europe & Central Asia | -6.6 (-8.0, -5.3) | -3.7 (-9.9, -0.8) | -2.4 (-6.2, 0.2) | -1.0 (-2.4, 0.2) | -5.5 (-7.2, -4.0) | -1.7 (-6.8, 0.5) | -0.5 (-4.6, 1.5) | -0.9 (-2.3, 0.5) |
| Latin America & Caribbean | -5.5 (-6.6, -4.5) | 0.005 (-2.8, 2.9) | 2.0 (-0.4, 3.6) | -0.7 (-1.8, 0.3) | -5.4 (-6.5, -4.1) | -1.0 (-3.5, 1.8) | 0.7 (-1.7, 3.0) | -1.0 (-2.1, 0.2) |
| North Africa and Middle East | -7.0 (-7.9, -5.9) | -3.8 (-9.7, -0.8) | -2.7 (-4.7, -0.5) | -1.3 (-2.5, -0.1) | -5.6 (-7.0, -4.2) | -1.8 (-6.7, 0.5) | -1.0 (-3.9, 1.0) | -1.1 (-2.4, 0.2) |
| South Asia | -5.0 (-5.9, -4.4) | -0.4 (-2.3, 1.1) | 1.0 (0.0, 1.8) | -1.6 (-2.3, -0.6) | -5.3 (-6.2, -4.4) | -0.7 (-2.5, 1.2) | 0.9 (-0.5, 2.3) | -1.5 (-2.4, -0.5) |
| Southeast Asia, East Asia, Oceania | -5.7 (-6.6, -4.8) | -1.8 (-3.6, -0.3) | 0.3 (-0.7, 1.1) | -1.0 (-1.5, -0.4) | -5.5 (-6.3, -4.7) | -1.5 (-3.3, -0.2) | 0.5 (-0.5, 1.4) | -1.1 (-1.7, -0.5) |
| Sub-Saharan Africa | -5.6 (-6.5, -4.6) | 0.1 (-1.4, 1.3) | 1.5 (0.6, 2.3) | -1.0 (-2.0, -0.2) | -5.5 (-6.7, -4.4) | -1.0 (-2.7, 0.8) | 0.7 (-0.7, 2.1) | -1.1 (-2.2, -0.1) |
